# Kondo-free mirages in elliptical quantum corrals

**DOI:** 10.1038/s41467-020-15137-8

**Published:** 2020-03-16

**Authors:** Qili Li, Xiaoxia Li, Bingfeng Miao, Liang Sun, Gong Chen, Ping Han, Haifeng Ding

**Affiliations:** 1 National Laboratory of Solid State Microstructures and Department of Physics, Nanjing University, 210093 Nanjing, China; 2 Collaborative Innovation Center of Advanced Microstructures, 210093 Nanjing, China; 3 School of Electronic Science and Engineering, Nanjing University, 210093 Nanjing, China

**Keywords:** Electronic devices, Surfaces, interfaces and thin films, Quantum mechanics

## Abstract

The quantum mirage effect is a fascinating phenomenon in fundamental physics. Landmark experiments on quantum mirages reveal atomic-scale transport of information with potential to remotely probe atoms or molecules with minimal perturbation. Previous experimental investigations are Kondo-effect based; the quantum mirages appear only near the Fermi energy. This strongly limits the exploration of the mechanism and potential application. Here we demonstrate a Kondo-free quantum mirage that operates in a wide energy range beyond Fermi energy. Together with an analytical model, our systematic investigations identify that the quantum mirage is the result of quantum interference of the onsite electronic states with those scattered by the adatom at the focus of elliptical quantum corrals, where two kinds of scattering paths are of critical importance. Moreover, we also demonstrate the manipulation of quantum mirages with pseudo basic logic operations, such as NOT, FANOUT and OR gates.

## Abstract (web summary)

The quantum mirage effect is a fascinating phenomenon but in general the underlying mechanism is unclear. Here, by building elliptical quantum corrals of adatoms on a metal surface, the authors establish the mechanism of Kondo-free mirages and utilize it to build atomic-scale logic gates.

## Introduction

A quantum corral is a closed nano-sized loop of atoms deposited on a substrate, as first constructed via scanning tunneling microscopy (STM) atomic manipulation by Crommie, et al.^1^. Quantum mirage is a term used to describe a fascinating phenomenon inside an elliptical quantum corral (EQC), when an atom is placed at one focal point, a mirage of the electronic properties of that atom appears at the other focus. The effect was first demonstrated in landmark experiments by Manoharan et al., where the Kondo resonance of a Co adatom placed at one of the foci of an EQC can be remoted imaged at the other focus^2^. Such mirages exhibit potential for remotely probing atoms or molecules with minimal perturbation^2^ and to enable transport of information at the atomic scale^3,4^.

Different theories^5–14^ reproduce the mirage effect, but point out different key factors for the formation mechanism. The theories tend to suggest that a high value of the local density of state (LDOS) at the focal positions is critical for the formation of quantum mirages^6–9^. Agam and Schiller, however, claimed that the phase accumulation in the quadrilateral path of the electron wavefunction scattering plays a key role while the ellipse eccentricity is not important^5^. The previous experiment is based on the Kondo effect, where the associated quantum mirages appear only near the Fermi energy^2^. This strongly limits the exploration of its mechanism and potential application. Thus, non-Kondo effect based and energy-dependent experimental investigations are highly desired. In addition, other interesting quantum mirages require experimental confirmation that have been predicted that are Kondo-free, such as the anti-mirage^15^, nonmagnetic mirage^15^, magnetic mirage^10,16,17^, mirages of a magnetic bound state on a superconductor and on a topological insulator^18,19^. Moreover, although pioneering experiments reveal transport of information^2^, the power of quantum mirages for advanced applications, such as logic operations at the atomic level^20,21^, have not yet been demonstrated.

Here, we demonstrate a Kondo-free quantum mirage that can operate in a wide energy range beyond the Fermi energy. By placing an Fe or Ag adatom at one of the foci of an EQC on a Ag(111) surface, we found that the LDOS atop the adatom can be probed in a wide energy range at the other adatom-free focal point. Our observations are Kondo-free because the Kondo effect neither appears in a wide energy range, nor occurs in the nonmagnetic Ag/Ag(111) system. Also, via systematic variation of the energy and the size of the EQC, we found the transfer function of the local density of states from target to probe depends on the wavenumber (*k*), major axis (*a*) and the eccentricity (*e*). We present an analytical model that yields quantitative agreement with experiment. The one-by-one correspondence identifies that quantum mirage is the result of quantum interference of the onsite electron states with those scattered by the adatom at one focal point of the EQC, where two types of scattering paths are of critical importance. Moreover, we also demonstrate the manipulation of the quantum mirage with basic logic operations, such as pseudo NOT, FANOUT, and OR gates.

## Results

### Inversion effect and Kondo-free quantum mirage

**Fig. 1:**
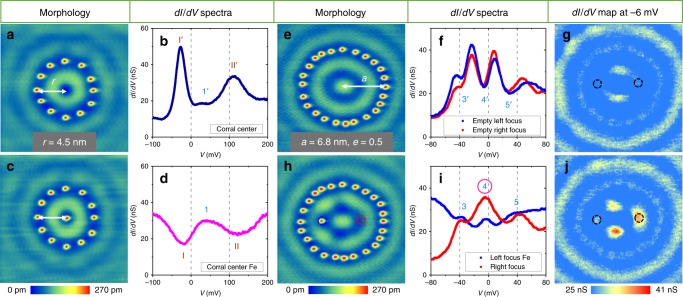
Inversion effect and quantum mirage. **a** Topography of a CQC (radius *r* = 4.5 nm) built with 12 Fe adatoms. **b** *dI/dV* spectrum obtained at the corral center with its peaks labelled by wine Roman numerals with apostrophe and a valley labelled by cyan Arabic numeral with apostrophe. **c** Topography of the same corral with an additional Fe adatom placed at the center. **d** *dI/dV* spectrum atop the center Fe adatom with its valleys labelled by wine Roman numerals and a peak labelled by cyan Arabic numeral. **e** Topography of an EQC (*a* = 6.8 nm and *e* = 0.5). **f** The corresponding *dI/dV* spectra obtained at the left focus (blue circle) and right focus (red circle) of the EQC. The valleys are labelled by cyan Arabic numerals with apostrophe. **g** *dI/dV* map of the EQC at −6 mV. **h** Topography of the same EQC but with an additional Fe adatom placed at the left focus. **i** *dI/dV* spectra obtained atop the Fe adatom placed at left focus (blue) and at the right focal position (red). The peaks are labelled by cyan Arabic numerals. **j** *dI/dV* map of the EQC in **h** at −6 mV. The dashed circles in **g**, **h**, **j** mark the focal positions of the EQC.

In our Kondo-free quantum mirage experiments, we utilized the electronic states caused by the inversion effect^22^, since they are localized states and exhibit strong oscillations in a wide energy range which are ideal for quantum mirage mechanism study. To illustrate the inversion effect, we show the topography of a circular quantum corral (CQC) constructed with 12 Fe adatoms (Fig. 1a) and its corresponding *dI/dV* spectrum obtained at the corral center (Fig. 1b). The spectrum has a peak-valley-peak feature (highlighted in the figure), which evolves to a valley-peak-valley with an Fe adatom at the center (Fig. 1c, d). The reversal of the spectral feature shows the inversion effect, which can be understood by the coupling between the adsorbate level and surface states of the substrate^22^ (Supplementary Note 1). The coupling reflects the broadening of the adsorbate state, which is proportional to the onsite LDOS of the surface states^22^. The higher the onsite LDOS of the surface states, the stronger the coupling, which results in a broadening and reduced intensity of the adsorbate state. In such a way, the features in the spectrum are reversed as compared to the one without an adatom. To further identify this, we performed similar measurements for CQCs with different diameters. The spectra obtained atop the adatoms placed at the corral centers all show inverted features as compared to the ones obtained at the center of the empty corrals of the same size (Supplementary Fig. 2), indicating that it is indeed an inversion effect.

Next we explore the effect of quantum mirages in EQCs (see topography in Fig. 1e), where the semi-major axis *a* = 6.8 nm and the eccentricity *e* = 0.5. The corresponding *dI/dV* spectra at left- and right-focus are almost identical, as expected from a symmetry point of view (Fig. 1f). The slight difference originates from small deviations of the adatoms from their ideal positions. The valleys are marked as 3′, 4′, and 5′. The *dI/dV* map at the sample bias of −6 mV (valley 4′) shows nearly no contrast at the left- and right-focus (Fig. 1g), consistent with the observed dip in the spectra. The two bright spots between the two foci represent the standing wave pattern of the empty corral. With an additional Fe adatom placed at the left focus (Fig. 1h), the measured spectrum atop it (blue curve in Fig. 1i) contains peaks 3, 4, and 5 originating from valleys 3′, 4′, and 5′ in Fig. 1f as caused by the inversion effect. Remarkably, the spectrum on the right focus (red curve in Fig. 1i) shares similar features with that of the Fe adatom at the left focus, indicative of the quantum mirage effect. Notably, the intensity of peak 4 at the right focus is even stronger than that of the Fe at the left focus with a ratio of ~1.4. The measured *dI/dV* map of the same area taken at −6 mV illustrates the quantum mirage spatially (Fig. 1j), where three bright spots and one faint spot are present in the corral. We find that the bright spot at the right focus is significantly brighter than the one at the left focus, which is consistent with the corresponding spectra. Since the inversion effect cannot be attributed to the Kondo effect, the observation of the bright spot at the right focus demonstrates a Kondo-free quantum mirage. The observed quantum mirage in Fig. 1j appears slightly larger than the feature at the left focus. This could be understood from a diffraction point of view. Here, the corresponding wavelength of the surface state at −6 mV is ~7.8 nm, which is much larger than the size of Fe atom at the left focus (~1 nm in diameter). Thus, we are essentially performing subwavelength imaging, and the image appears larger than the object and slightly distorted. When we increased the energy for imaging, the distortion reduced, as shown in Supplementary Figs. 3b, h and 4b. Note that the two middle spots are the standing wave pattern as also observed in the empty corral shown in Fig. 1g. The small horizontal offset of the two middle spots between panel g and j of Fig. 1 is due to the in-corral adatom perturbation^23^.

To further explore the Kondo-free quantum mirage, we performed several controlled experiments. First, we followed the previous method^2^ and moved the Fe adatom away from the focus by ~1 nm or placed it at other high symmetry points and found that the quantum mirage effect disappeared (Supplementary Figs. 3 and 4), indicating the importance of the focal positions. Second, we shifted the additional Fe adatom to the right focus instead of the left focus of EQC and found that the quantum mirage remained unchanged within our experimental error margin (Supplementary Figs. 5 and 6). Third, we replaced the focus Fe adatom by a Ag adatom, and observed a comparable quantum mirage (Supplementary Fig. 7). This further supports that our observation is indeed a Kondo-free quantum mirage since Ag/Ag(111) is nonmagnetic.

### Mechanism of Kondo-free quantum mirage

**Fig. 2:**
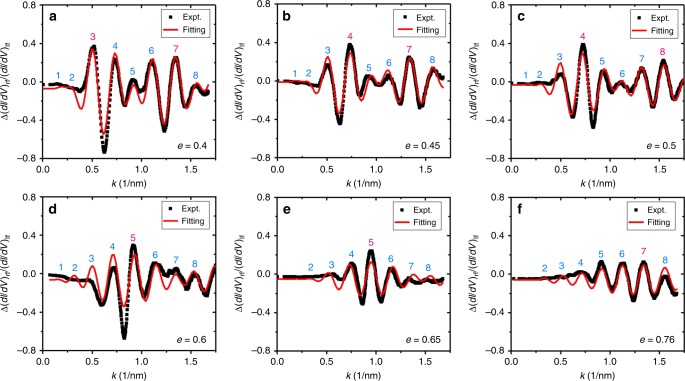
*e*-dependent transfer function. **a**–**f** The transfer function of quantum mirage in EQCs with fixed *a* = 7.6 nm and *e* = 0.4–0.76, as indicated. Black squares are the experimental data, and red curves are the fittings utilizing Eq. (2). The peaks are marked by numbers defined in Eq. (3). The magenta numbers are distinguished from the cyan ones to mark the central peaks. The values of wavenumber *k* are obtained using the dispersion relation discussed in main text.

Next, we quantitatively investigated the influence of an Fe adatom at the left focus on the spectrum at the unoccupied right focus for a series of the EQCs (Supplementary Figs. 8 and 9). In Fig. 2, we plot the transfer function, *i.e*. ${\mathrm{\Delta }}\left( {dI/dV} \right)_{{\mathrm{rf}}}/\left( {dI/dV} \right)_{{\mathrm{lf}}}$ as a function of *k* for EQCs with fixed *a* = 7.6 nm and various *e* values. $\Delta \left( {dI/dV} \right)_{{\mathrm{rf}}}/\left( {dI/dV} \right)_{{\mathrm{lf}}}$ refers to the difference of the right focus spectrum between cases with and without the Fe atom at the left focus, normalized by the spectrum obtained atop the Fe adatom at the left focus. The wavenumber *k* is obtained via the dispersion relation of the surface state, namely $E = \frac{{{\hbar}^{2} k^2}}{{2m^ \ast }} + E_0$ with m^*^ = 0.42*m*_e_ and *E*_0_ = −65 meV^24^. All of the transfer functions show strong oscillations with *k* within the range of −0.8 to 0.5 and the peak positions exhibit an approximate linear *k*-dependence. The oscillating amplitude is *k*-dependent and the overall features present modulated oscillations, suggesting that the transfer functions are modulated by two different periods. In addition, the transfer function is also *e*-dependent. In order to better describe the spectra, we locate the central peak (peak number highlighted in magenta) which has a greater magnitude than its neighboring peaks, i.e. peaks 3 and 7 are the central peaks at *e* = 0.4 (Fig. 2a). With increasing *e*, the central peaks move toward higher *k* values, i.e. the central peaks shift to peaks 4 and 8 at *e* = 0.5 (Fig. 2c). The movement of the central peaks indicates that one of the modulations is *e*-dependent. Moreover, we also found that the amplitude of the transfer-function oscillation gradually decreases from *e* = 0.4 to *e* = 0.7, suggesting an *e*-dependent decay.

**Fig. 3:**
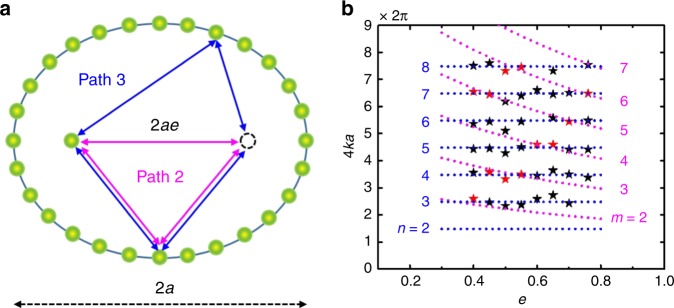
Schematics of the quantum interference model and the quantization conditions. **a** Sketch of an EQC with different scattering paths. **b** Comparison of the experimentally determined peak positions (red stars are the central peaks and the black stars are the rest peaks) and the theoretically obtained quantized conditions. Blue dotted lines marked by *n* are the condition corresponding to Path 3 (blue quadrangle in **a**) described in Eq. (3), and magenta dotted curves marked by *m* are the conditions corresponding Path 2 (magenta triangle in **a**) described in Eq. (4).

To understand the interesting dependence, we derived a Green’s functions based analytical model^5,11,17,25–28^ (see Methods). Figure 3a shows a sketch of possible scattering paths for an EQC with an adatom placed at the left focus. Generally, we consider the quantum interference between the onsite electron states with those scattered by the adatom placed at the left focus. By taking into account different paths, the variation of LDOS at the right focus induced by the adatom at the left focus can be obtained as:

1 $$\Delta \rho _{{\mathrm{rf}}}\left( k \right) = \rho _{{\mathrm{lf}}}\mathop {\sum}\limits_{i = 1}^l {A_i} {\mathrm{cos}}\left( {kd_i + \delta _i} \right){\mathrm{exp}}\left[ { - \frac{{d_i}}{{2L_\phi \left( k \right)}}} \right],$$

where Δ*ρ*_rf_ represents the change of LDOS at the adatom-free right focus caused by placing an adatom at the left focus position and *ρ*_1f_ is the LDOS atop the adatom at the left focus; *A*_*i*_, *d*_*i*_ and *δ*_*i*_ stand for the scattering amplitudes, the lengths of the path, and the phase shifts in different paths. *L*_*ϕ*_(*k*) is the *k*-dependent phase coherence length^29^. Due to the observed decay behavior, we only consider three shortest scattering paths. Path 1 (not shown): the straight path for the electron states directly bounced back by the adatom at the left focus. Path 2 (magenta triangular in Fig. 3a): the triangle path where the scattering of the electron wavefunction occurs at the adatom at the left focus subsequently at the corral circumference or vice versa, then back to the right focus. Path 3 (blue quadrangle in Fig. 3a): the quadrilateral path corresponds to the electron states scattered sequentially by the corral, adatom at the left focus and corral again, then back to the right focus. Therefore, the length of the path can be obtained as: $d_1 = 4ae$, $d_2 = 2a\left( {1 + e} \right)$ and *d*_3_ = 4*a*. The phase shifts can be obtained as $\delta _1 = \delta _{\mathrm{f}}$, $\delta _2 = \delta _{\mathrm{f}}{\mathrm{ + }}\delta _{\mathrm{c}} + \frac{\pi }{4}$ and $\delta _3 = \delta _{\mathrm{f}}{\mathrm{ + 2}}\delta _{\mathrm{c}} - \frac{\pi }{2}$ (Methods), where *δ*_c_ and $\delta _{\mathrm{f}}$ are the phase shifts via the scattering by the corral and the adatom located at the focus, respectively. According to the number of the scattering and the length of the path, the scattering amplitudes along the different paths can be calculated as $A_1 = - \rho _{\mathrm{s}}{\mathrm{\Delta }}_{\mathrm{s}}\sqrt {1 + \left[ {\cot \left( {\delta _{\mathrm{f}}} \right)} \right]^2} /\left( {kae} \right)$, $A_2 = 2\pi ^{7/2}\eta \alpha \rho _{\mathrm{s}}^{2}{\mathrm{\Delta }}_{\mathrm{s}} \sqrt {1 + \left[ {\cot \left( {\delta _{\mathrm{f}}} \right)} \right]^2} /\left( {ka\sqrt {kae} } \right)$ and $A_{3} = {\eta} ^{2}\pi^{7}\alpha^{2}{\rho}_{\mathrm{s}}^{3} {\mathrm{\Delta }}_{\mathrm{s}}\sqrt {1 + \left[ {\cot \left( {\delta _{\mathrm{f}}} \right)} \right]^2} /\left( {ka} \right)^2$. Here Δ_s_ is the hybridization energy of the adatom at the left focus and surface state. The factor *η* comes from the sum term of single scattering, which is about 0.9 here (see Methods and Supplementary Fig. 10a). As Path 1 requires strict backscattering which has a very limited scattering angle, it is anticipated that *A*_1_ is much smaller than *A*_2_ and *A*_3_ (see the calculated ratio in Supplementary Fig. 10b). From the calculation, we also find that *A*_2_ is comparable with *A*_3_. Thus, the transfer function can be approximated as follows:

2 $$\frac{{{\mathrm{\Delta }}\left( {dI/dV} \right)_{{\mathrm{rf}}}}}{{\left( {dI/dV} \right)_{{\mathrm{lf}}}}} \,\approx	 \, \frac{{{\mathrm{\Delta }}\rho _{{\mathrm{rf}}}}}{{\rho _{{\mathrm{lf}}}}} \approx A_2\cos \left[ {2\left( {1 + e} \right)ka + \delta _2} \right]\exp \left[ { - \frac{{a\left( {1 + e} \right)}}{{L_\phi \left( k \right)}}} \right] \\ 	+ A_3\cos \left( {4ka + \delta _3} \right)\exp \left[ { - \frac{{2a}}{{L_\phi \left( k \right)}}} \right].$$

Equation 2 contains two quantization conditions. One corresponds to the second term, namely

3 $$4ka + \delta _3 = 2n\pi .$$

This condition results in the oscillations of the quantum mirage with *a*, as was observed by Manoharan et al.^2^ and then explained theoretically^5,7^. The second one, which has not been reported before, corresponds to the first term and has the form:

4 $$2\left( {1 + e} \right)ka + \delta _2 = 2m\pi .$$

We note that the first term is *e*-dependent, which is consistent with our experimental findings.

To validate the model, we fitted our experimental results using Eq. (2). As shown in Fig. 2, the theoretical curves reproduce the experimental data in all cases. In Eq. (2), $L_\phi \left( k \right) = \frac{{{\hbar}^{2}}}{{2m^ \ast }}\frac{k}{{{Im} \Sigma }}$ contains the imaginary part of the self-energy ${Im} \Sigma = \gamma + \beta \left( {E - E_F} \right)^2$^30^, where *γ* is a constant inversely proportional to the lifetime of surface states scattering between intra-surface band or inter-bulk band, and the quadratic term describes the Fermi liquid behavior. *β* is typically close to zero^29^, and the energy range is close to the Fermi energy. Thus, we treat Im∑ ≈ γ and use it as a fitting parameter. The fitting yields *γ* = 9.7 ± 0.7 meV, which is larger than the value of 4.9 ± 0.6 meV obtained on the defect-free surface state^31^, suggesting that the scattering by the corral adatoms may reduce the lifetime of the surface state. The phase shifts obtained are *δ*_c_ = (1.12 ± 0.03)π and *δ*_f_ = (1.25 ± 0.09)π (see Supplementary Table 1 for the details). The amplitude related products are $\alpha \rho _{\mathrm{s}} = 0.28 \pm 0.03$ and $\Delta _{\mathrm{s}}\rho _{\mathrm{s}} = 0.19 \pm 0.02$. The small error margins for all these parameters in different EQCs prove the validity of the fitting.

We also crosschecked the aforementioned quantization conditions. Figure 3b shows the theoretically derived quantization conditions and the experimentally determined peak positions, where all peaks agree with the theoretical quantization conditions. Interestingly, the central peaks are located at the cross points of two theoretically derived conditions, suggesting that both quantization conditions are of critical importance for the high-intensity quantum mirage. The one-to-one correspondence between the peak positions and the quantization conditions supports the validity of the quantum interference model.

**Fig. 4:**
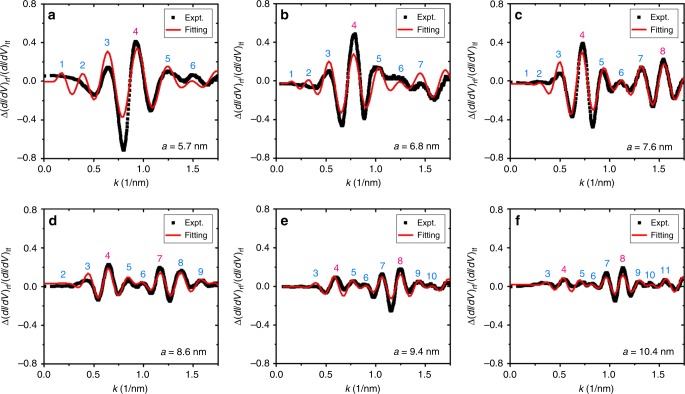
*a*-dependent transfer function. **a**–**f** The transfer function of quantum mirage in EQCs with fixed *e* = 0.5 and *a* = 5.7–10.4 nm, as noted. Black squares are the experimental data, and red curves are the fittings utilizing Eq. (2). The peaks are marked by numbers defined in Eq. (3).

To further test the model, we also performed an *a*-dependent study for EQCs with fixed *e* = 0.5. Figure 4 shows the experimentally obtained *a*-dependent transfer functions. Once again, the theoretical fittings agree with the experimental data. The parameters obtained of *γ* = 8.0 ± 0.3 meV, *δ*_c_ = (1.12 ± 0.03)π, *δ*_f_ = (1.38 ± 0.07)π, $\alpha \rho _{\mathrm{s}} = 0.27 \pm 0.01$ and $\Delta _{\mathrm{s}}\rho _{\mathrm{s}} = 0.16 \pm 0.01$ are consistent with the values obtained in our *e*-dependent study (see Supplementary Table 2 for the details). By crosschecking the two quantization conditions in the *a*-dependent study, we found that the experimental peaks are in a good agreement with these conditions and the central peaks only appear at the cross points of the two conditions (Supplementary Fig. 11). By studying the *a*-dependent amplitude, we also found two different decay behaviors (Supplementary Fig. 12), which correspond to the different lengths of the paths. The ratio between the characteristic lengths of these two decay behaviors is 1.2 ± 0.1, which is consistent with the expected ratio of 2/(1 + *e*) for *e* = 0.5. This demonstrates that the decay behaviors are indeed dominated by the aforementioned Paths 2 and 3 in Fig. 3a. With the derived transfer function, we can compute the *dI/dV* spectra that appears at the right focus. The results are in good agreement with the experimentally obtained spectra (Supplementary Fig. 13). We note that our model also provides an explanation of previous Kondo effect based quantum mirage experiment^2^. In it, a striking feature was found: a large change in amplitude but almost no change in width in the projected Kondo resonance peak. This can be understood in that the Kondo resonance only appears in a narrow energy window near the Fermi energy where the transfer function is almost a constant. Thus, the transfer function has little influence on the peak width of the projected Kondo resonance.

### Manipulation of quantum mirage and pseudo logic operations

**Fig. 5:**
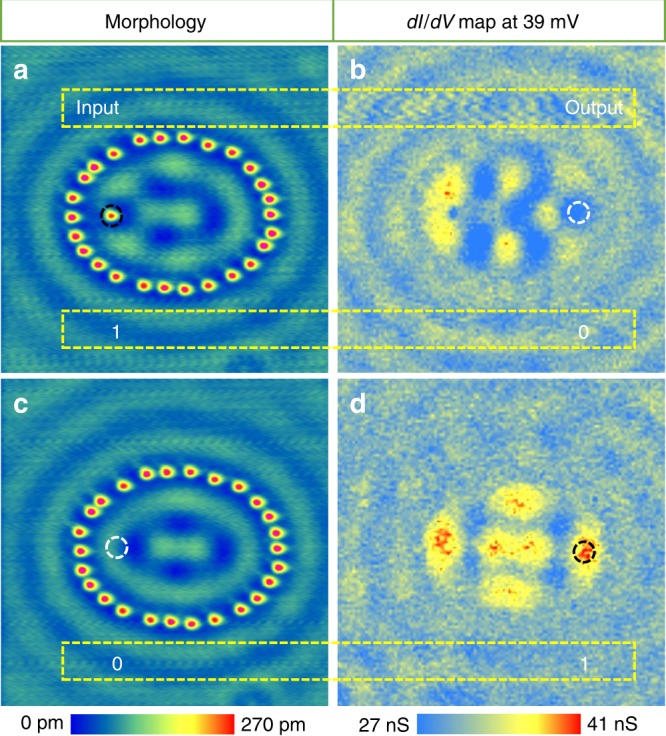
Inverted mirage and pseudo NOT gate. **a** Topography for an EQC (*a* = 7.6 nm and *e* = 0.65) with an Fe adatom placed at the left focus. **b** The corresponding *dI/dV* map at the bias voltage of +39 mV for the EQC in **a**. **c** Topography of the EQC without the Fe adatom at the left focus. **d** The corresponding *dI/dV* map with the same bias voltage of +39 mV for the empty EQC in panel c. Dashed circles mark the focal positions, for which black means 1 and white means 0.

In the following, we discuss the manipulation of quantum mirages and potential applications. In a quantum mirage, the constructive phase is used to achieve the high-intensity mirage. Quantum interference may also lead to destructive interference when an additional π-phase shift is introduced, e.g. the transfer functions at the dip positions in Fig. 2 have negative values. The destructive interference can be used to manipulate the mirage to an inverted mirage (anti-mirage) as well as to construct a pseudo NOT gate. For instance, the presence/absence of the adatom at one focus as the input “1”/“0” may trigger a high/low difference of *dI/dV* intensity at the other focus as the output “1”/“0”^3^. We, thus, define this type of operation as pseudo logic, which is demonstrated in an Fe adatom based EQC (Fig. 5). In the presence of the Fe adatom at the left focus (Fig. 5a), the corresponding *dI/dV* map of the EQC at +39 mV (corresponding to the valley position between peak 5 and 6 in Fig. 2e) shows a dark contrast at the right focus (Fig. 5b), demonstrating an inverted mirage (anti-mirage). When the Fe adatom is removed from the focus (Fig. 5c), the corresponding *dI/dV* map at the same energy shows a bright spot at the right focus (Fig. 5d). The results are summarized in Table 1. The presence/absence of the Fe adatom at the left focus (input “1”/“0”) induces a significant difference in the *dI/dV* intensity at the right focus (output “0”/“1”), which satisfies the function of a NOT gate.

**Table 1:** The relation between the output and input for pseudo NOT gate.

Input (Atom occupation)	Output (*dI/dV* in nS)
1	27.2 ± 1.1
0	38.7 ± 1.6

**Fig. 6:**
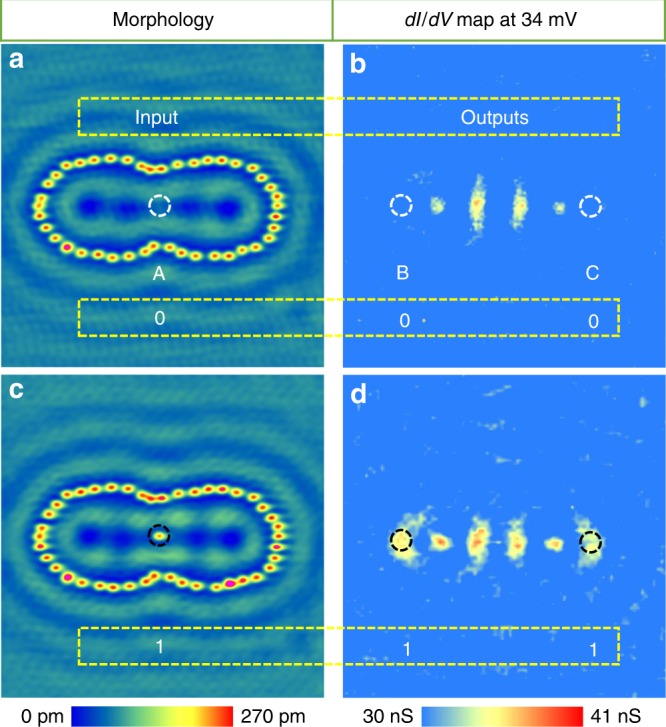
Pseudo FANOUT gate. **a** Topography of a confocal EQC (both the EQCs used to build the confocal EQC have the same size of *a* = 6.6 nm and *e* = 0.7). The joint focus A is used as the input and the other two foci B and C are the outputs. **b** The corresponding *dI/dV* map of panel a at the bias voltage of +34 mV. **c** Topography of the same confocal EQC in panel a but with an additional Fe adatom placed at A. **d** The corresponding *dI/dV* map of **c** at the same bias voltage of +34 mV. Dashed circles mark the focal positions, for which black means 1 and white means 0.

The number of degrees of freedom of the proposed logic devices is two in an EQC system because EQC has two foci, which is only applicable for a two-terminal device. To realize multi-terminal devices, we design a special geometry by combining two EQCs with one joint focus to form a confocal EQC (Fig. 6a). The three foci (one joint focus A and the other two individual foci B and C) form a three-terminal platform for the logic device design. The concept of a pseudo FANOUT gate is illustrated in Fig. 6. We control the presence/absence of an adatom at joint focus A as the input “1”/“0” and *dI/dV* intensity at B and C as the output. The *dI/dV* map obtained from “0” input (Fig. 6a) at a bias voltage of +34 mV shows low contrast at both foci B and C (*dI/dV* values of 30.2 and 31.7 nS, respectively) (Fig. 6b), corresponding to “0” outputs. When an Fe adatom is placed at the joint focus A (Fig. 6c), the corresponding *dI/dV* map (Fig. 6d) show high intensity (*dI/dV* values of 35.6 and 35.2 nS, respectively which correspond to “1” in output) at both outputs. The relations between the output and input are summarized in Table 2, and it shows a pseudo FANOUT logic gate. When we swap the input and output, a pseudo OR logic gate can also be constructed (Supplementary Fig. 14 and Supplementary Table 3).

**Table 2:** The relation between the outputs and input for pseudo FANOUT gate.

Input A (Atom occupation)	Output B (*dI/dV* in nS)	Output C (*dI/dV* in nS)
0	30.2 ± 1.0	31.7 ± 1.2
1	35.6 ± 1.4	35.2 ± 0.8

Here, as a prototype device, the logic threshold of the on-off signal of ~1.5 is demonstrated. It is somewhat less than that of the real devices^32^. This ratio, however, can be improved via several approaches. For instance, in our work, the bulk state contributes as a constant background that significantly reduces the on-off ratio. Thus, a higher ratio can be achieved if the experiment is performed on a system with a reduced or even vanishing bulk state. Besides, as above discussed, the amplitude of the transfer function depends on the DOS of the surface state and the hybridization energy. When the intensities of these two terms are increased, a higher ratio is also expected.

### Summary

We demonstrate a Kondo-free quantum mirage that can operate in a wide energy range. Via systematic size and energy-dependent studies, we show that the transfer function is a modulated oscillation function which is (*k, a, e*)-dependent. In combination with a Green’s-function-based analytical approach, our experiments reveal the physical mechanism of a quantum mirage, which is the quantum interference of the onsite electronic states with those scattered by the adatom and the corral. We found two major scattering paths connecting two foci are of critical importance, a tetragonal one with two bounds at the corral and a triangle one with one bound at the corral. Harnessing the quantum interferences, we also demonstrated a two-terminal pseudo NOT logic gate and a three-terminal pseudo FANOUT and OR logic gates. Our results open a playground for the design and exploration of the atomic logic devices. In addition, in our logic gates, essentially only the wave property of the electrons is used. This kind of wave property, such as the standing wave inside a corral, is also realized in optical systems^33^. Thus, the demonstrated concepts are expected to be transferrable to other wave-based devices, such as optical or magnon devices^34,35^.

## Methods

### Experimental details

Experiments were performed in a low-temperature STM system with base pressure 2 × 10^−11^ mbar. The temperature for STM/STS measurements was ~4.7 K. The Ag(111) single crystal was cleaned by cycles of sputtering and annealing. High-purity Fe was deposited onto the Ag(111) surface with a typical rate of 0.002 monolayer equivalent per minute by means of electron beam evaporation at ∼6 K for several seconds. The Ag atom was obtained by means of the atom transfer technique^36^. The tip was stabilized at *I* = 1 nA and *V*_bias_ = 50 mV, which refers to the sample voltage with respect to the tip. STS measurements were performed using a lock-in technique with the modulation of the sample voltage of 4 mV at a frequency of 6.3 kHz. The lateral atom manipulation^37–39^ was used to build corrals.

### Derivation of the analytical model

We consider the system by the Hamiltonian $H = H_0 + H_{{\mathrm{imp}}}$, where $H_0 = \mathop {\sum}\nolimits_{\mathbf{k}} {\varepsilon _{\mathbf{k}}c_{\mathbf{k}}^\dagger c_{\mathbf{k}}}$ and $H_{{\mathrm{imp}}} = \sum_{i = 0}^{N} \sum_{\mathbf{k}} [ \varepsilon_{\mathrm{a}} a_{i}^{\dagger} a_{i} + \left( V_{\mathrm{s}} c_{\mathbf{k}}^{\dagger} a_{i} + H.C.\right)]$ describe the free surface-state electrons and adatoms on the surface, respectively. For *H*_imp_, we further decompose it into two parts: (i) *i* ≠ 0, adatoms form the ellipse corral; (ii) *i* = 0, the adatom locates within the ellipse corral. Note that the spin index is omitted for simplification. Firstly, we consider the ellipse corral formed by *N* atoms (*i* ≠ 0). The Green’s function in the presence of the corral can be expanded according to the scattering times as^5^

5 $$G_{\mathrm{c}}\left( {{\mathbf{r}},{\mathbf{r}}^{\prime} ;E} \right) = G_0\left( {{\mathbf{r}},{\mathbf{r}}^{\prime} ;E} \right) + G_1\left( {{\mathbf{r}},{\mathbf{r}}^{\prime} ;E} \right) + G_2\left( {{\mathbf{r}},{\mathbf{r}}^{\prime} ;E} \right) + \ldots ,$$

where $G_0\left( {{\mathbf{r}},{\mathbf{r}}^{\prime} ;E} \right) = - {\boldsymbol{i}}\pi \rho _{\mathrm{s}}H_0^{(1)}\left( {k\left| {{\mathbf{r}} - {\mathbf{r}}^{\prime} } \right|} \right)$ is the free two-dimensional Green’s function^5,27^. *ρ*_s_ is surface-state density of states. $H_0^{(1)}\left( x \right)$ is the zeroth-order Hankel function of the first kind, which takes the asymptotic form $H_{0}^{(1)} (x) \approx \sqrt {\frac{2}{{\pi x}}} \exp \left( {{\boldsymbol{i}} x - {\boldsymbol{i}}\frac{\pi }{4}} \right)$ for *x* » 1^5^. $G_1\left( {{\mathbf{r}},{\mathbf{r}}^{\prime} ;E} \right) = \mathop {\sum}\nolimits_{j = 1}^N {G_0( {{\mathbf{r}},{\mathbf{R}}_j;E} )} s_i\left( E \right)G_0 ( {{\mathbf{R}}_j,{\mathbf{r}}^{\prime} ;E} )$ is the sum of all the one-time scattering. In it, $s_i\left( E \right) = \alpha \left( E \right)e^{{\boldsymbol{i}}\delta _{\boldsymbol{c}}\left( E \right)}$ is the adatom *s*-wave transition matrix, with energy-dependent amplitude *α* and phase shift *δ*_c_. $G_2\left( {{\mathbf{r}},{\mathbf{r}}^{\prime} ;E} \right) = \mathop {\sum}\nolimits_{i,j = 1}^N {G_0\left( {{\mathbf{r}},{\mathbf{R}}_i;E} \right)} s_i\left( E \right)G_0\left( {{\mathbf{R}}_i,{\mathbf{R}}_j;E} \right)s_j\left( E \right)G_0\left( {{\mathbf{R}}_j,{\mathbf{r}}^{\prime} ;E} \right)$ is the sum of all the two-time scattering. Followed high order terms are the sum of the entire three-time scattering, four-time scattering and so on.

Secondly, we consider another adatom within the corral (*i* = 0). We treat the whole scatterings of the corral as the background, and now the total Green’s function for this system is below^26^

6 $$G_{\mathrm{t}}\left( {{\mathbf{r}},{\mathbf{r}}^{\prime} ;E} \right) = G_{\mathrm{c}}\left( {{\mathbf{r}},{\mathbf{r}}^{\prime} ;E} \right) + G_{\mathrm{c}}\left( {{\mathbf{r}},{\mathbf{R}};E} \right)\left| {V_{\mathrm{s}}} \right|^2G_{\mathrm{a}}\left( E \right)G_{\mathrm{c}}\left( {{\mathbf{R}},{\mathbf{r}}^{\prime} ;E} \right).$$

The influence of the extra adatom to the corral $\Delta G_{\mathrm{t}}\left( {{\mathbf{r}},{\mathbf{r}}^{\prime} ;E} \right) = G_{\mathrm{t}}\left( {{\mathbf{r}},{\mathbf{r}}^{\prime} ;E} \right) - G_{\mathrm{c}}\left( {{\mathbf{r}},{\mathbf{r}}^{\prime} ;E} \right)$ by substituting Eq. (5) into Eq. (6) is as below

7 $${\Delta G_{\mathrm{t}}\left( {{\mathbf{r}},{\mathbf{r}}^{\prime} ;E} \right)} 	= \, {\left( {G_0 + G_1 + G_2 + \ldots } \right)\left| {V_{\mathrm{s}}} \right|^2G_{\mathrm{a}}\left( E \right)\left( {G_0 + G_1 + G_2 + \ldots } \right)} \\ 	= \, G_0\left| {V_{\mathrm{s}}} \right|^2G_{\mathrm{a}}\left( E \right)G_0 + G_0\left| {V_{\mathrm{s}}} \right|^2G_{\mathrm{a}}\left( E \right)G_1 + G_1\left| {V_{\mathrm{s}}} \right|^2G_{\mathrm{a}}\left( E \right)G_0 \\ 	\,\,\,\,\,\,\,\,+ G_1\left| {V_{\mathrm{s}}} \right|^2G_{\mathrm{a}}\left( E \right)G_1 + \ldots .$$

To make comparisons with the experimental results, we concentrate on the interferences at the right focus induced by left focus Fe adatom. The Green’s function at unoccupied right focus is

8 $$\Delta G_{\mathrm{t}}\left( {{\mathbf{R}},{\mathbf{R}};E} \right) = 	\, G_0\left| {V_{\mathrm{s}}} \right|^2G_{\mathrm{a}}\left( E \right)G_0 + G_0\left| {V_{\mathrm{s}}} \right|^2G_{\mathrm{a}}\left( E \right)G_1 + G_1\left| {V_{\mathrm{s}}} \right|^2G_{\mathrm{a}}\left( E \right)G_0 \\ 	+ G_1\left| {V_{\mathrm{s}}} \right|^2G_{\mathrm{a}}\left( E \right)G_1 + \ldots .$$

The first term is the direct path between the two foci. The second and third terms are the triangular paths shown in Fig. 3a. The fourth term is the quadrangular path as shown in Fig. 3a. Note that the hybridization energy between the adatom and surface state is $\Delta _{\mathrm{s}}={\boldsymbol{\pi }}\rho _{\mathrm{s}}\left| {V_{\mathrm{s}}} \right|^2$. The free two-dimensional Green’s function between the two foci is: $G_0\left( {{\mathbf{R}}, - {\mathbf{R}},E} \right) = G_0\left( { - {\mathbf{R}},{\mathbf{R}},E} \right) = - {\boldsymbol{i}}\rho _{\mathrm{s}}\sqrt {\frac{\pi }{{kae}}} \exp \left( {{\mathrm{i}}2kae - {\mathrm{i}}\frac{\pi }{4}} \right)$, and is short for $G_0 = - {\mathrm{i}}\rho _{\mathrm{s}}\sqrt {\frac{{\boldsymbol{\pi }}}{{kae}}} \exp \left( {{\mathrm{i}}2kae - {\mathrm{i}}\frac{\pi }{4}} \right)$ hereinafter. Then the one-time scattering Green’s function is $G_1 = - \alpha \rho _{_{\mathrm{s}}}^2\mathop {\sum}\nolimits_{j = 1}^N {\frac{\pi }{{k\sqrt {\xi _1\xi _2} }}\exp \left( {{\mathrm{i}}2ka + {\mathrm{i}}\delta - {\mathrm{i}}\frac{\pi }{2}} \right)}$. *ξ*_1_ and *ξ*_2_ are the distance connecting the right focus with corral wall and corral wall with left focus. The sum can be numerically calculated as $\mathop {\sum}\nolimits_{j = 1}^N {\frac{1}{{k\sqrt {\xi _1\xi _2} }}} = \mathop {\sum}\nolimits_{j = 1}^N {\frac{1}{{k\sqrt {\left( {a - ex_j} \right)\left( {a + ex_j} \right)} }}} = \mathop {\sum}\nolimits_{j = 1}^N {\frac{1}{{ka\sqrt {\left\{ {1 - e\cos \left[ {2{\boldsymbol{\pi }}\frac{{\left( {j - 1} \right)}}{N}} \right]} \right\}\left\{ {1 + e\cos \left[ {2\pi \frac{{\left( {j - 1} \right)}}{N}} \right]} \right\}} }} = } \frac{{\eta \left( {e,N} \right)\pi ^3}}{{ka}}$, with factor *η*(*e*, *N*) related to *e* and *N*. We note that the atoms’ number *N* used to build corrals is varied from 24 to 26 to keep the mean distance between adjacent adatoms unchanged. *η* is almost a constant of 0.9 in the EQCs that were investigated (see Supplementary Fig. 10).

Thirdly, we move on to the LDOS. The variation of LDOS is obtained as:

9 $$\Delta \rho \left( {{\mathbf{R}},E} \right) = - \frac{1}{{\boldsymbol{\pi }}}{Im} \left[ {\Delta G_{\mathrm{t}}\left( {{\mathbf{R}},{\mathbf{R}},E} \right)} \right].$$

In addition, the lifetime of surface states is taken into account by an additional decay of the electron amplitude as $\exp ( { - r/L_\phi })$^29,40,41^. Finally, we reach the result of Eq. (1) in the main text. Note that although we did not consider bulk states in the Hamiltonian, their influence on surface states can been incorporated into the lifetime of surface states by inter-band scattering. In addition, an alternative approach that includes the influence of bulk states is also feasible by considering the imaginary part of the phase shift^6,11,42^.

## Supplementary information

Supplementary Information

Peer Review File

## Data Availability

The data that support the plots within this paper and other findings of this study are available from the corresponding authors upon request.
