# Supplementary material for: Kondo-free mirages in elliptical quantum corrals
Source: Nat Commun. 2020 Mar 16;11:1400. doi: 10.1038/s41467-020-15137-8 (PMC7075878; doi:10.1038/s41467-020-15137-8)
Supplement: Supplementary file 1 — Supplementary Information [file 41467_2020_15137_MOESM1_ESM.pdf]

Supplementary Information

Kondo-free mirages in elliptical quantum corrals

Li et al.

Supplementary Note 1: Bound states of Fe and Ag adatoms on Ag(111) (Supplementary Fig. 1);

Supplementary Note 2: Inversion effect for CQCs of different radii (Supplementary Fig. 2);

Supplementary Note 3: Importance of focal position for quantum mirages in EQCs (Supplementary Figs. 3, 4);

Supplementary Note 4: Symmetry check for Fe adatom placed at left or right focus (Supplementary Figs. 5, 6);

Supplementary Note 5: Quantum mirage for a Ag adatom in an EQC (Supplementary Fig. 7);

Supplementary Note 6: Quantum mirages of different-sized EQCs (Supplementary Figs. 8, 9);

Supplementary Note 7: Computed η and relative ratio for the scattering intensity among different paths (Supplementay Fig. 10);

Supplementary Note 8: Fitting parameters for changing e (Supplementary Table 1);

Supplementary Note 9: Fitting parameters and quantization conditions for changing a (Supplementary Fig. 11, Supplementary Table 2);

Supplementary Note 10: Decay behavior check (Supplementary Fig. 12);

Supplementary Note 11: Comparison of the computed the dI/dV spectra at the right focus with the one obtained experimentally (Supplementary Fig. 13);

Supplementary Note 12: Pseudo OR logic gate (Supplementary Fig. 14, Supplementary Table 3).

Supplementary Note 1: Bound states of Fe and Ag adatoms on Ag(111)

Supplementary Fig. 1a shows the *dI/dV* map of an isolated Fe adatom on a wide Ag(111) terrace at a bias voltage of −130 mV. It exhibits high intensity at the adatom position while rapidly decaying away from the adatom (see the line profile in the inset at the top right). This demonstrates the existence of a localized state. The corresponding topographic image is inserted at the left bottom. The *dI/dV* spectrum on top of the Fe adatom is shown in Supplementary Fig. 1b, which presents a resonance peak at −130 meV . The resonance is assigned to the adatom-induced bound state[1-3], resulting from the coupling of the Fe adatom's *s* state with the surface states[4, 5]. It was suggested that the resonance is irrelevant with the *d*-state magnetism of the Fe adatom[4, 5].

To confirm this, we performed experiments with a Ag adatom placed on a wide Ag(111) terrace, and observed a similar bound state. Supplementary Fig. 1c shows the *dI/dV* map of a single Ag adatom at a bias voltage of −100 mV. The spatial distribution demonstrates it a localized state. The corresponding topographic image is inserted at the left bottom. The Ag atom was obtained by means of the atom transfer technique[6]. Firstly, a W tip was decorated with Ag atoms by soft indentations into the surface. Secondly, a feedback loop was open after the tip was stabilized at V_{bias} = 120 mV, I = 1 nA. Thirdly, the tip was driven towards the surface by 0.5 nm with rate ~0.1 nm·s^{-1} and retracted back at the same rate. Finally, we scanned the area to check if there was tip-apex atom transfer. The Ag atom was further identified by the bound state[3]. The *dI/dV* spectrum over the Ag adatom is presented in Supplementary Fig. 1d, which is consistent with the work of Ref.[3].

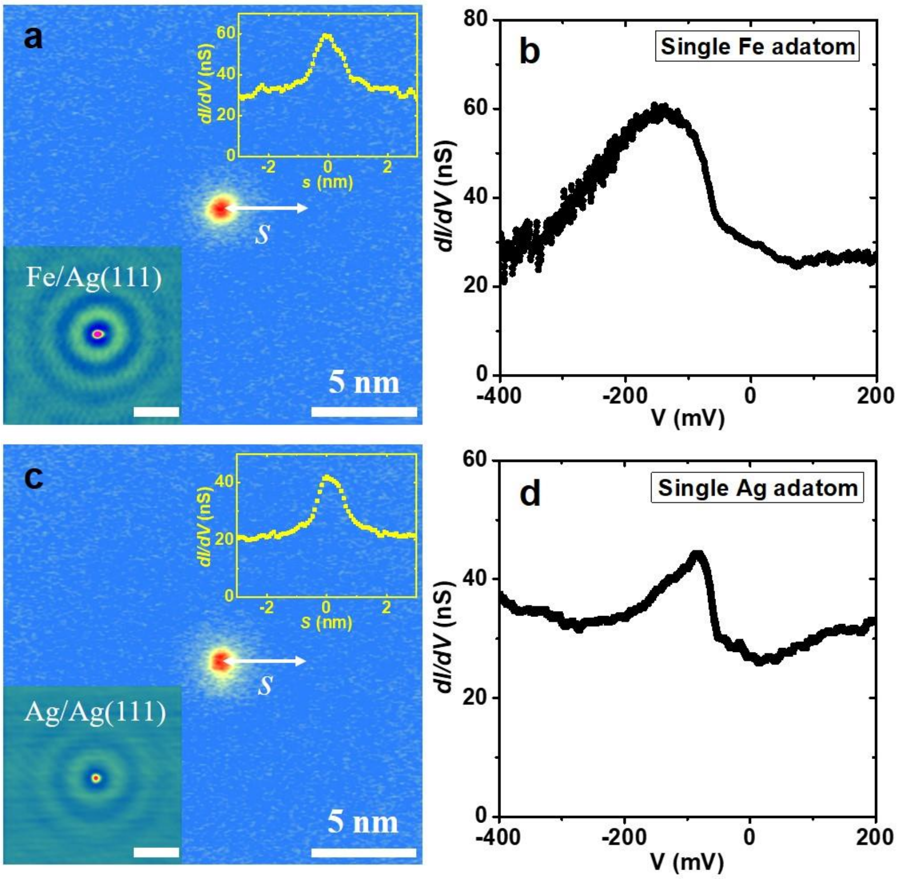

Supplementary Figure 1 | Bound states of Fe and Ag adatoms on Ag(111). a, *dI/dV* map at a bias voltage of -130 mV of an Fe adatom on a wide Ag(111) terrace showing the spatial distribution of the bound state. Inset at the top right shows the line profile across the adatom center. Inset at the bottom left shows the corresponding topographic image. **b**, *dI/dV* spectrum of a single Fe adatom on Ag(111). It shows a resonance peak at -130 mV. **c**, *dI/dV* map at -100 mV of a Ag adatom on a wide Ag(111) terrace. Inset at the top right shows the line profile across the adatom center. Inset at the bottom left shows the corresponding topographic image. **d**, *dI/dV* spectrum of a single Ag on Ag(111), which shows a resonance peak around -100 mV.

Supplementary Note 2: Inversion effect of CQCs of different radii

Here, we clarify the inversion effect for circular quantum corrals of different radii. Supplementary Fig. 2a shows a typically topographic image of an empty corral built with Fe adatoms. When the corral is small (radius $r = 3.5$ nm), there is only one quantum well state (peak I´) probed at the center of the empty corral in the measured voltage range (Supplementary Fig. 2b). When enlarging r to 5.5 nm, three quantum well states emerge as peaks I´, II´ and IIII´ (Supplementary Fig. 2c). After placing single Fe adatoms at the centers of the corresponding corrals (Supplementary Fig. 2d), the spectra obtained on top of the Fe adatom at the corral center are shown in Supplementary Figs. 2e and 2f, respectively. These spectra display one-to-one inverted features of those of the empty corrals (peak to dip and dip to peak). This demonstrates that the observed effect is indeed an inversion effect.

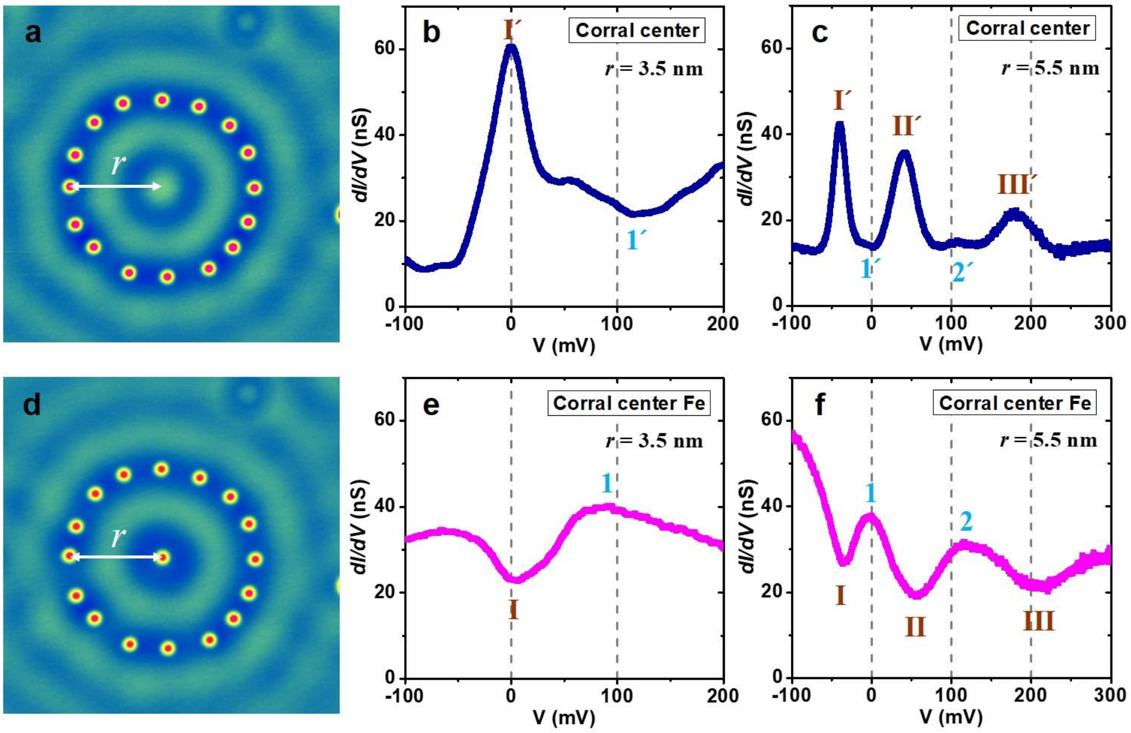

Supplementary Figure 2 | Inversion effect for CQCs of different radii. a, Typical topographic image of empty circular corrals. **b**, **c**, *dI/dV* spectra obtained at the centers

of the empty corrals with r = 3.5 nm and 5.5 nm, respectively. **d**, Typical topographic image of an Fe adatom placed at the center of the same corral of panel a. **e**, **f**, *dI/dV* spectra obtained on top of the Fe adatom placed at the corral centers with r = 3.5 nm and 5.5 nm, respectively. The tip was stabilized at V_{bias} = 50 mV, I = 1 nA for STM imaging and *dI/dV* spectra measurements (hereinafter the same unless specified).

Supplementary Note 3: Importance of focal position for quantum mirages in EQCs

Supplementary Fig. 3 illustrates the importance of the focal position for quantum mirages in EQCs with two examples. For this purpose, we compared the *dI/dV* map for the Fe adatoms placed at the focal position, slightly off the focal position and the empty EQC. Supplementary Fig. 3a shows the topographic image of an EQC (e = 0.6, a = 7.6 nm) with an extra Fe adatom placed at the left focus. The corresponding *dI/dV* map at a bias voltage of +15 mV is shown in Supplementary Fig. 3b. We find a bright spot at the right focus (Supplementary Fig. 3b), demonstrating the quantum mirage effect. With the interior Fe atom moved slightly off of focus (Supplementary Fig. 3c), the bright spot at that right focus disappears (Supplementary Fig. 3d), indicating the important role of the focal position for the presence of quantum mirages. Supplementary Fig. 3e is the image with the interior Fe atom removed. For the empty corral, there are also no bright spots at the two foci, while the bright spots between two foci remain (Supplementary Fig. 3f). Note that the pattern near the center of the EQC is caused by quantum interference, as it can be found in all three cases. Supplementary Figs. 3g-3l reveal essentially the same effect but with EQCs with a different e value (= 0.65), once again proving the important

role of the focal position.

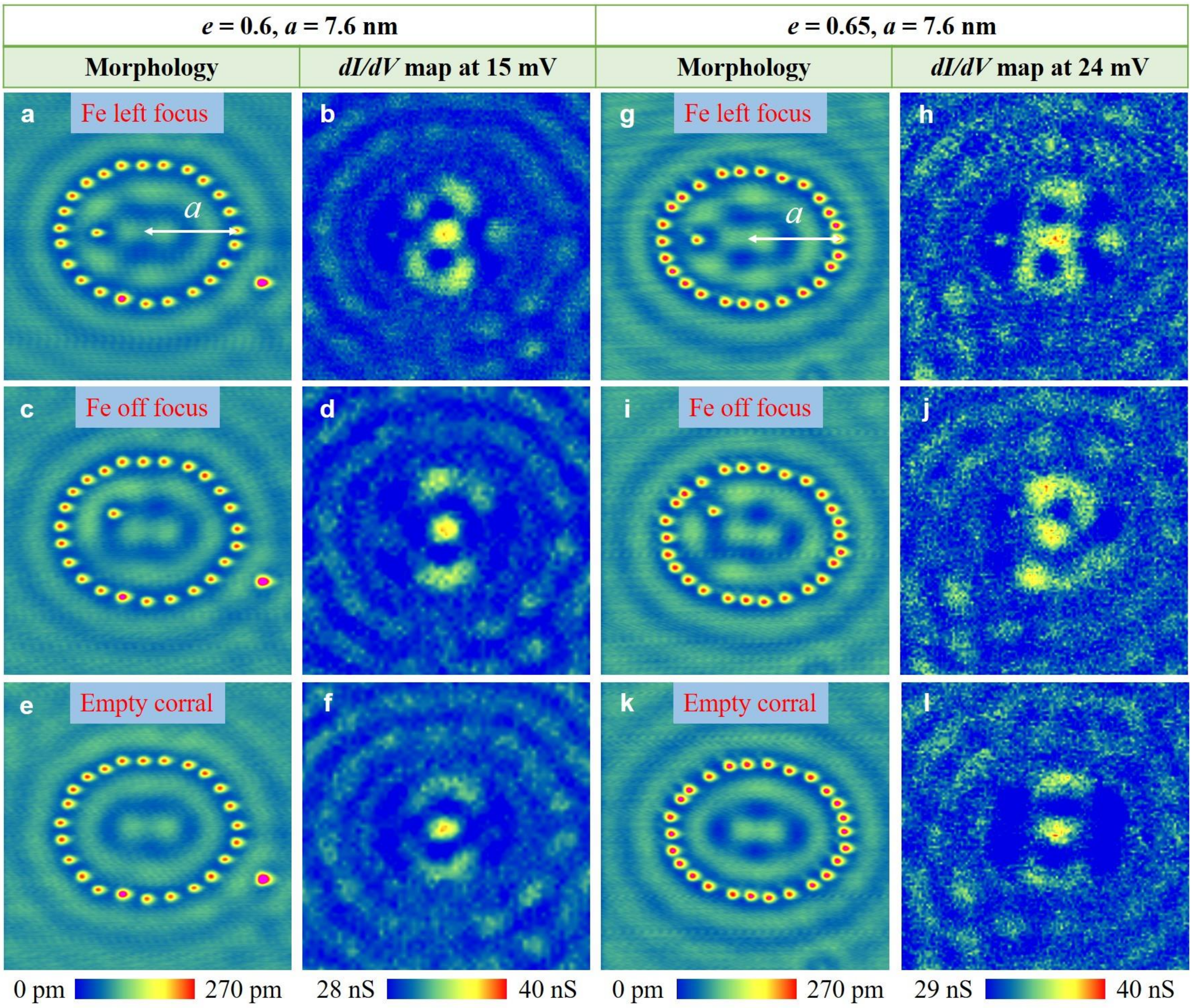

Supplementary Figure 3 | Comparison of the *dI/dV* maps for the addition Fe adatom placed at different positions for two EQCs of different size. a, **c**, **e** are the topographic images of an EQC (e = 0.6, a = 7.6 nm) for Fe left focus, Fe off focus, and an empty corral, respectively. **b**, **d**, **f** are the corresponding *dI/dV* maps at 15 mV. **g**, **i**, **k** are the topographic images of another case with e = 0.65, a = 7.6 nm for Fe left focus, Fe off focus, and empty corral, respectively. **h**, **j**, **l** are the corresponding *dI/dV* maps at 24 mV.

We also performed the *dI/dV* spectral analysis at high symmetry points of the ellipse. More specifically, we placed the adatom at different points, either at the long axis or the short axis, and made spectroscopy measurements. The results are shown below in Supplementary Fig. 4. From the figure, we see that the quantum mirage is only obtained when the adatom is placed at the focus position (Supplementary Figs. 4a and 4b). When the adatom is not at the focal point, even though it is at a high symmetry point of the ellipse, there is no spectral feature apparent either at the focal point or its symmetrical point (Supplementary Figs. 4c-4h). This once again confirms the importance of the focal point, and the effect that we observe is indeed the quantum mirage.

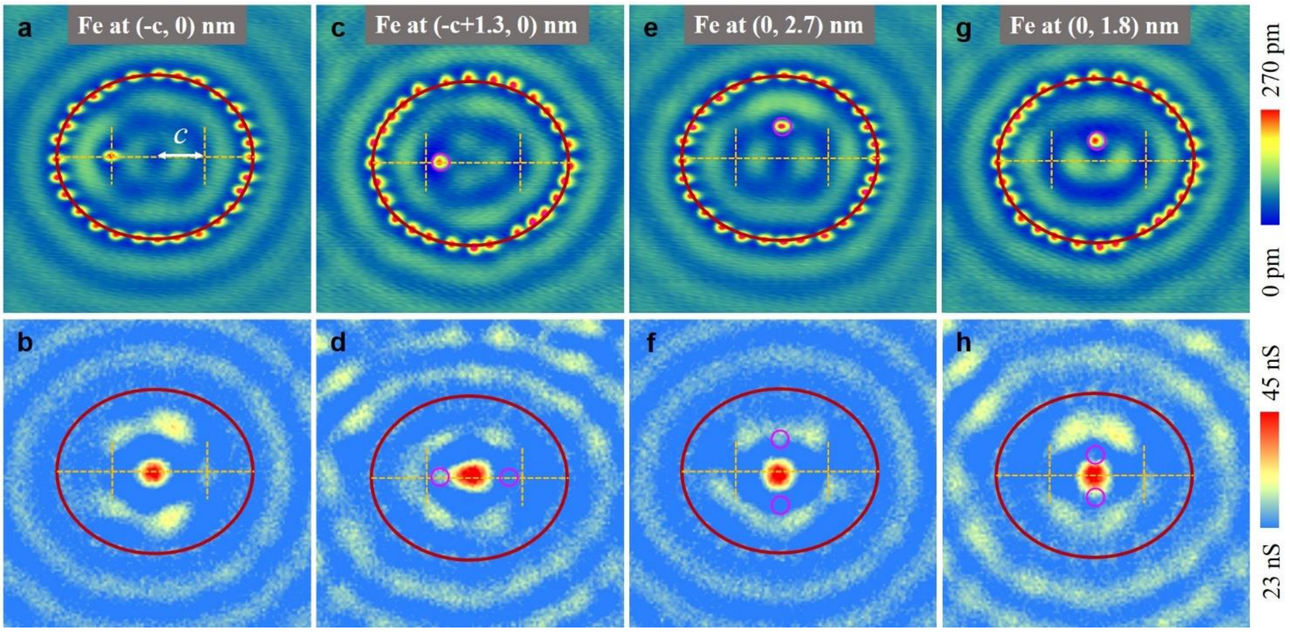

Supplementary Figure 4 | Comparisons of *dI/dV* map for the adatoms placed at different locations of an EQC with $e = 0.5$, $a = 7.8$ nm. The *dI/dV* maps were measured at 10 mV. The origin of coordinates is the ellipse center. The red ellipses, yellow crosses and purple circles mark the positions of the quantum corrals, the foci and the adatoms and their corresponding symmetrical points, respectively.

Supplementary Note 4: Symmetry check for Fe adatom placed at left or right focus

To explore the influence of the symmetry on the quantum mirage, we performed control experiments for Fe adatom at left or right focus. Supplementary Fig. 5a shows the topographic image of an EQC (e = 0.5 and a = 7.6 nm) with an Fe adatom placed at the left focus. The measured *dI/dV* spectra of both the left focus Fe adatom (blue curve) and right focus (red curve) are presented in Supplementary Fig. 5b. The spectrum on the right focus (red curve in Supplementary Fig. 5b) mimics that of the Fe adatom at left focus (blue curve in Supplementary Fig. 5b), indicating the quantum mirage effect. When moving the extra Fe adatom to the right focus (Supplementary Fig. 5c), the spectrum on the right focus (red curve in Supplementary Fig. 5d) also resembles that of the Fe adatom at left focus. The peak 4 still has the strongest intensity (blue curve in Supplementary Fig. 5d). Notice the similarity of the spectra for the Fe adatom placed at the left and the right foci, indicating that the symmetry of the Fe adatom positioning (at the left or right focus) has little influence on the quantum mirage. Any difference is attributed to slight differences of background spectra between left and right focus (Supplementary Fig. 5f), which is caused by the imperfect symmetry for the atom positioning in constructing the EQC (Supplementary Fig. 5e).

To obtain the influence of the extra Fe adatom on the quantum mirage, we subtract the corresponding spectrum of the empty corral and normalize it by the spectrum on the Fe adatom. Supplementary Fig. 6 shows the transfer functions for Fe adatom at left focus (Supplementary Fig. 6a) and right focus (Supplementary Fig. 6b). They exhibit oscillations, where labels 3 to 6 denote the peaks. The peaks of Supplementary Fig. 6a

have one-to-one correspondence with that of Supplementary Fig. 6b. The transfer functions for Fe at the left focus or right focus are almost the same. The only difference is in their intensities. Therefore, imperfections of EQCs in our experiments have little influence on our results.

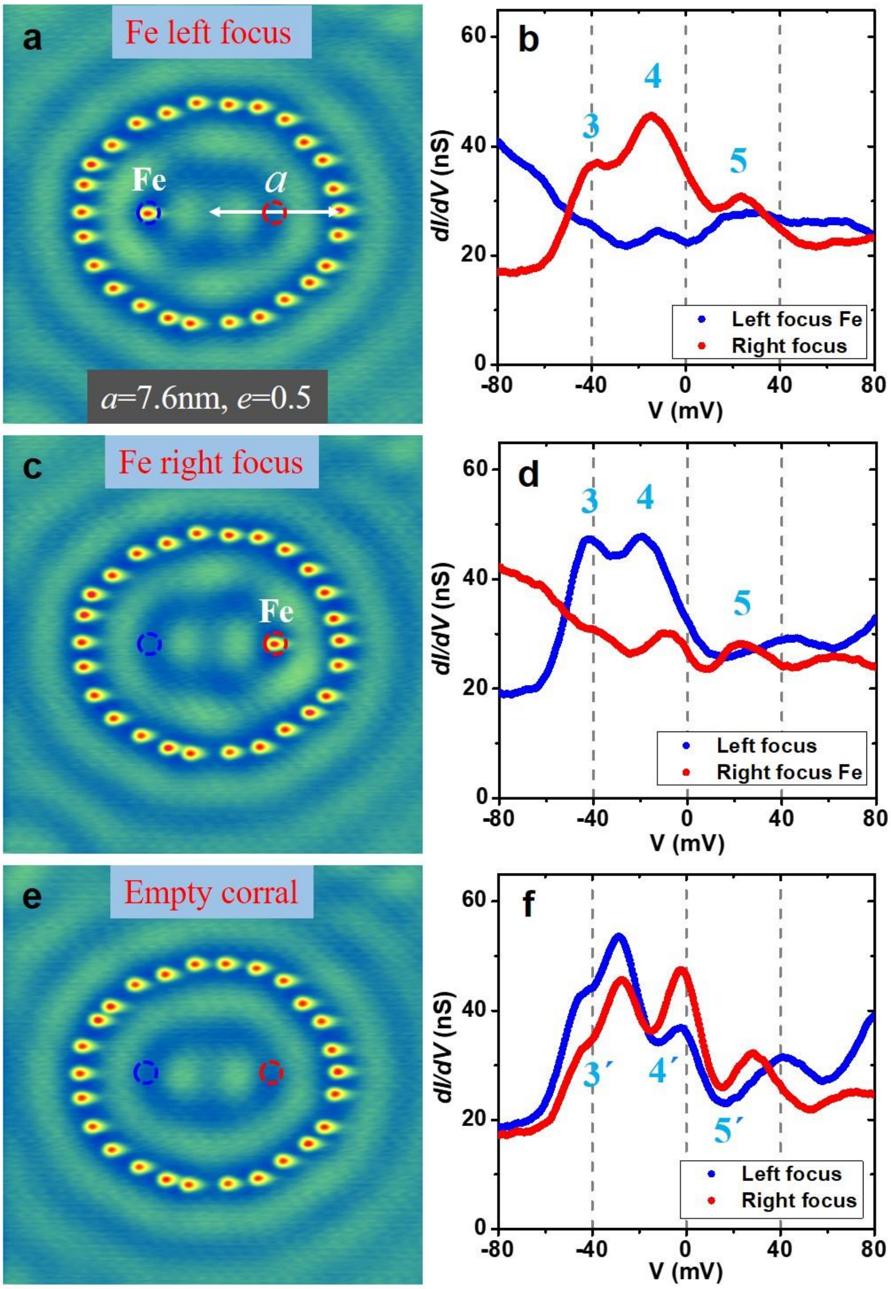

Supplementary Figure 5 | Quantum mirages for Fe adatom at left focus and right focus. a, **c**, **e**, The topographic images of an EQC with $e = 0.5$, $a = 7.6$ nm for Fe at left

focus, right focus, and empty corral, respectively. **b**, **d**, **f**, The corresponding spectra for Fe at left focus, right focus, and empty corral, respectively.

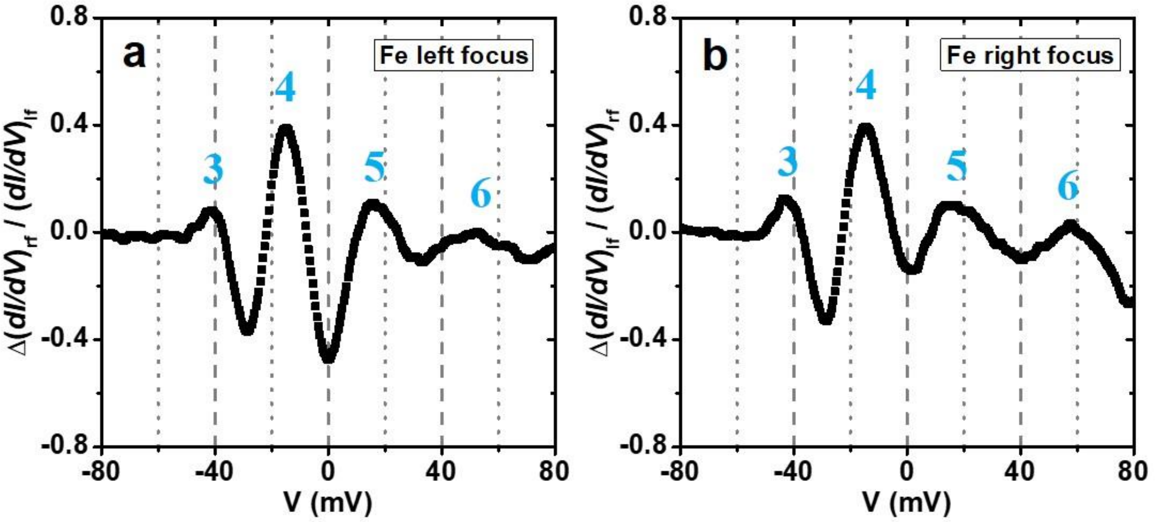

Supplementary Figure 6 | Transfer function for Fe adatom placed at left or right focus. a, for Fe adatom at left focus. **b**, for Fe adatom at right focus. The transfer functions are almost the same.

Supplementary Note 5: Quantum mirage for a Ag adatom in an EQC

The inversion effect results from the hybridization of the adatom's *s* state with the surface states. Therefore, the inversion effect induced quantum mirage is irrelevant to magnetism. To test this statement experimentally, we performed the following experiments with a (nonmagnetic) Ag atom. Firstly, we built the corral atom-by-atom with Fe atoms. Supplementary Fig. 7a shows the topograph of an EQC ($e = 0.5$, $a = 7.6$ nm) with a Ag adatom at the left focus. Note that a Ag atom was transferred to the corral by the aforementioned atom-transfer technique and crosschecked with the identified bound state. The measured spectra over the left focus Ag adatom (blue curve) and right focus (right focus) are presented in Supplementary Fig. 7b. The peaks at the left focus

Ag and right focus (labelled 3 - 6) line up with one-to-one correspondence. This indicates the transfer of the electronic structure of the left focus Ag adatom to the right focus. Interestingly, peak 4 of the right focus is much stronger than that of the left focus Ag (Supplementary Fig. 7b), which is the same behavior we observed with an Fe adatom. Supplementary Fig. 7c shows the corresponding transfer function. It is almost the same with that of Fe adatom (Supplementary Fig. 6), which further attests to the generality of the quantum mirage concept.

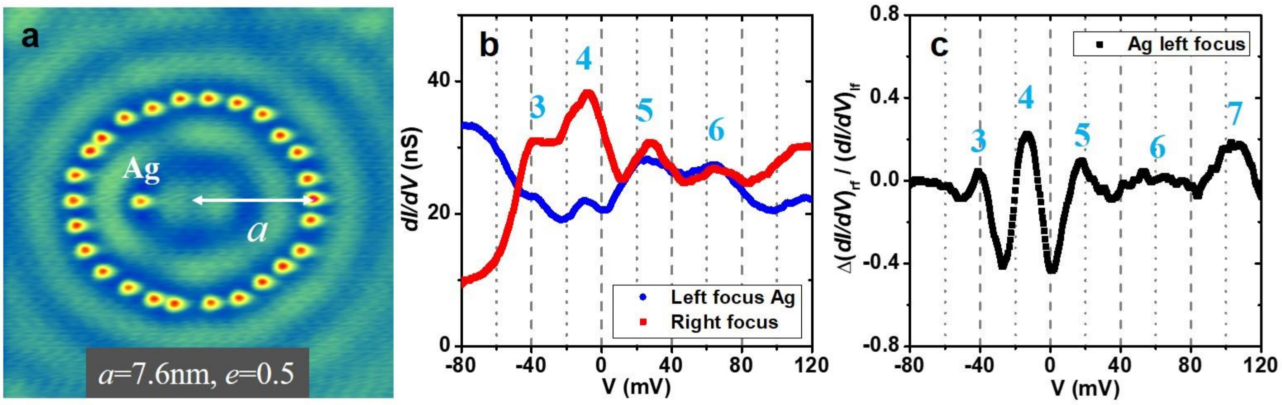

Supplementary Figure 7 | Quantum mirage and transfer function for Ag adatom in an EQC. a, An EQC with a = 7.6 nm, e = 0.5. **b**, The spectra on the left focus Ag adatom (blue curve) and right focus (red curve). **c**, The transfer function from the left focus Ag adatom to the right focus.

Supplementary Note 6: Quantum mirages of different-sized EQCs

In this part, we further demonstrate the generality of quantum mirages. For an EQC, there are two-independently controllable parameters. First, we performed experiments for EQCs with a fixed a and different e values. Then, we performed the experiments for EQCs with different a values and a fixed e.

Supplementary Fig. 8 presents typical *dI/dV* spectra for EQCs with a fixed *a* and different *e* values. Supplementary Fig. 8a shows the topographic image of an EQC (*a* = 7.6 nm, *e* = 0.5) with an Fe atom located at the left focus. Supplementary Fig. 8b is the *dI/dV* spectrum obtained on top of the Fe adatom located at the left focus. It exhibits peaks labelled 3, 4 and 5. The spectrum obtained at the right focus is present in Supplementary Fig. 8c. It also shows several peaks with the peak positions lining up with those in Supplementary Fig. 8b. Peak 4 in Supplementary Fig. 8c is much stronger than that of Supplementary Fig. 8b, confirming the results found in Fig. 1 of our paper. They are similar with those of Figs. 1f and 1i, while the peaks shift left. The stronger intensity in peak 4 reflects the high signal intensity, which differs from that of an attenuated Kondo resonance[7]. We also find that other peaks, like 3 and 5, are not enhanced. Thus, the enhancement is energy-dependent. Supplementary Fig. 8d presents the topographic image of an EQC with the same *a* value but a larger eccentricity, namely *e* = 0.6. Supplementary Figs. 8e and 8f are the spectra obtained on top of the left focus Fe adatom and at the right focus, respectively. Besides peaks 3, 4 and 5, another peak (labelled 6) emerges. These peaks line up with each other in Supplementary Figs. 8e and 8f, respectively. When further changing the eccentricity to 0.76 (Supplementary Fig. 8g), the spectra on the left focus Fe adatom and right focus are displayed in Supplementary Figs. 8h and 8i, respectively. The peaks marked 3 to 7 in both figures, again line up with each other.

Supplementary Fig. 9 shows the *a*-dependent (*e* = 0.5) quantum mirages. Supplementary Figs. 9a, 9d and 9g are the topographic images with *a* = 6.8, 7.6, 9.4 nm,

respectively. The corresponding spectra on left focus Fe (blue curves) and right focus (red curves) are presented in Supplementary Figs. 9b, 9e, 9h and 9c, 9f, 9i, respectively. The corresponding peaks shift toward the left as the value of a increases. In addition, the amplitude of the peaks decreases (or decay) with increasing a. For a quantitative analysis, see the discussions below.

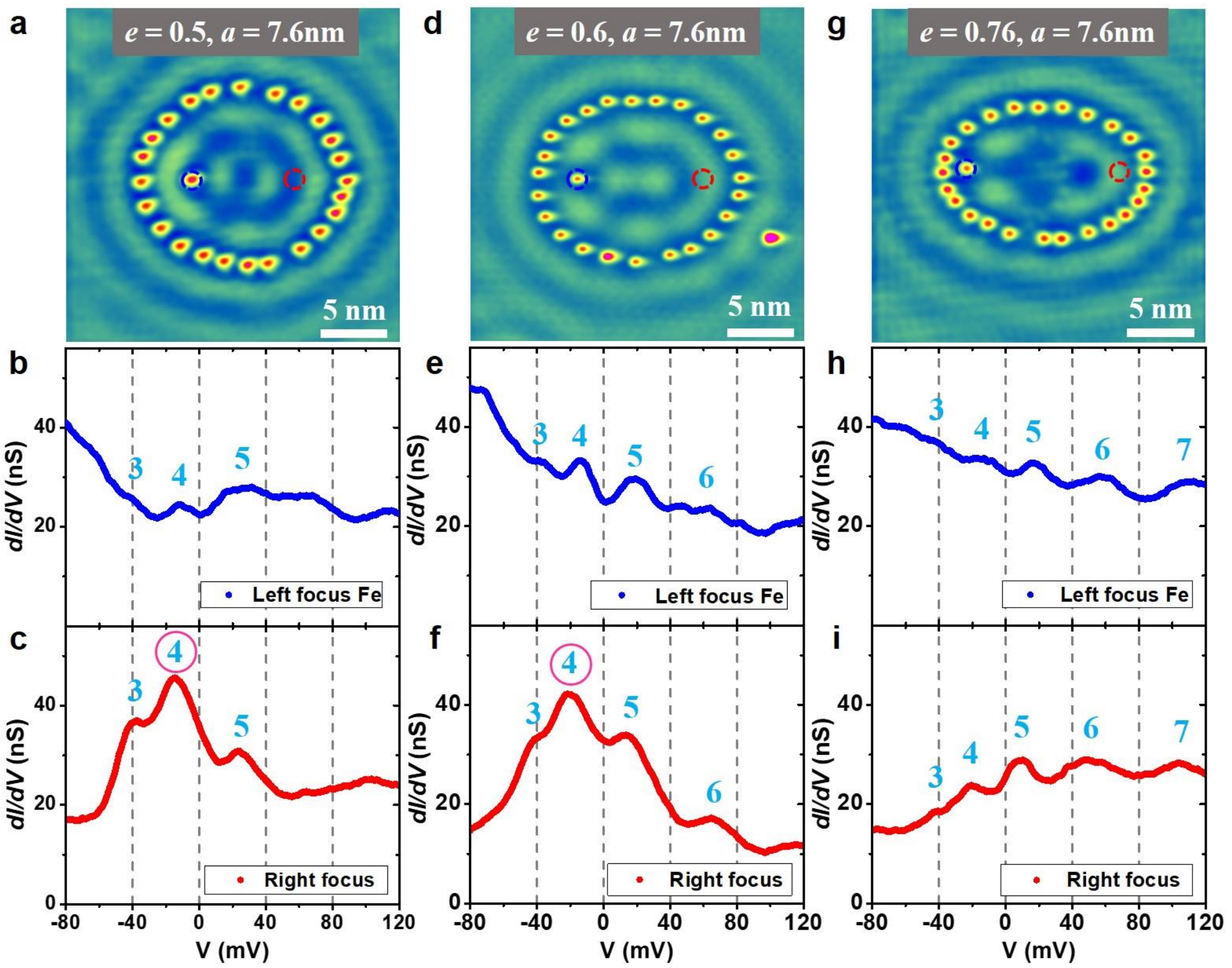

Supplementary Figure 8 | Quantum mirages for EQCs with fixed *a* and different *e* values. a, **d**, **g** are topographic images of the EQCs with the same a = 7.6 nm but different values of e = 0.5, 0.6, 0.76, respectively. Blue and red dotted circles are used to mark the left and right focus, respectively. Blue curves in **b**, **e**, **h** are the spectra obtained on top of the Fe adatom placed at the left focus of the corresponding EQCs.

Red curves in **c**, **f**, **i** are the spectra obtained at corresponding right focus. The numbers label the peaks.

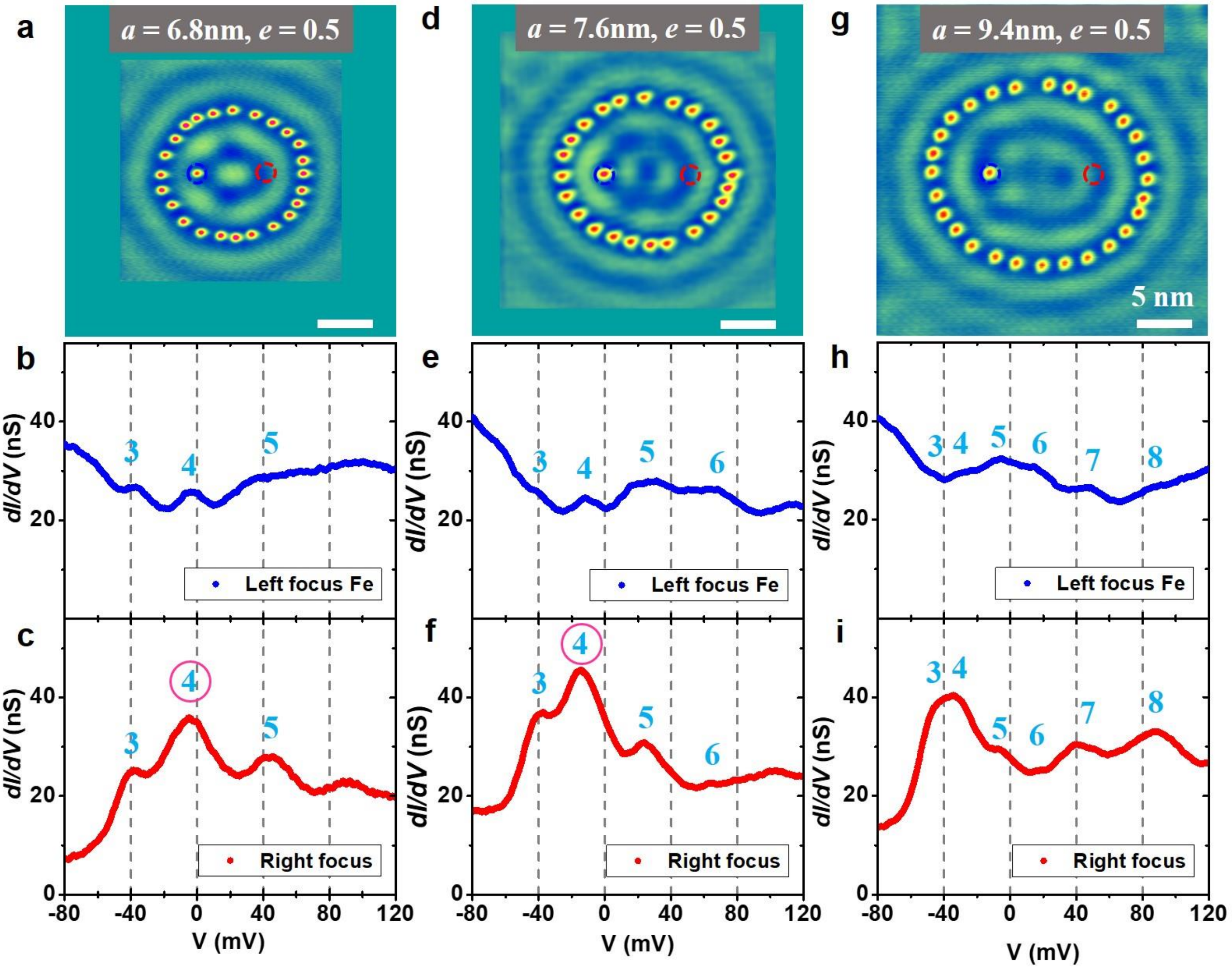

Supplementary Figure 9 | Quantum mirages for EQCs with different *a* and fixed *e* values. **a**, **d**, **g** are topographic images of EQCs with the same e = 0.5 and different values of a = 6.8, 7.6, 9.4 nm, respectively. The blue and red dotted circles mark the positions of the left and right focus, respectively. Blue curves in **b**, **e**, **h** and red curves in **c**, **f**, **i** are spectra on top of the Fe adatom placed at the left focus and at right focus position, respectively. The numbers label the peaks.

Supplementary Note 7: Computed η and relative ratio for the scattering intensity among different paths.

The factor η describes the sum term of single scattering. Supplementary Fig. 10a shows the value of η versus *e* for different *N*. The number of atoms *N* used to build the corrals is varied from 24 to 26 to keep the mean distance between adjacent adatoms unchanged. Note that η is almost around 0.9 in the range of *e* experimentally studied. Supplementary Fig. 10b shows the ratio of the scattering amplitudes for the first three terms with k = 0.83 nm^{-1} (Fermi wave number) and a = 7.6 nm. Note that A_2/A_3 is comparable, while A_1/A_3 is very small. Thus, the dominant terms are A_2 and A_3.

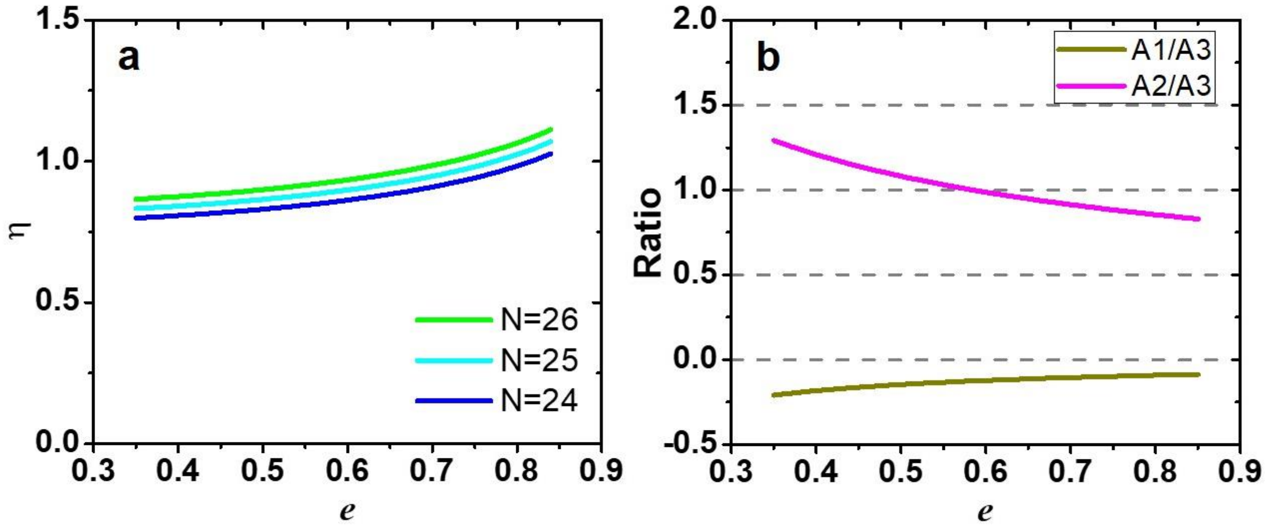

Supplementary Figure 10 | Caculated model parameters. a, The value of η. **b**, The relative ratio of the scattering amplitudes among the three different paths.

Supplementary Note 8: Fitting parameters for changing the value of *e*

We used Eq. (2) to fit the experimental results of Fig. 2 in main text. The values obtained for these parameters appear in Supplementary Table 1. Note that parameters δ_c,

δ_f and γ can be obtained directly from the fittings, while only the products $\alpha\rho_s$ and $\Delta_s\rho_s$ can be similarly obtained. To extract the values of α and Δ_s, the surface-state density of states ρ_s is needed.

Supplementary Table 1 Fitting results obtained for changing the value of *e* (*a* = 7.6 nm). δ_c and δ_f are the phase shifts for an adatom that is either forming the corral or sitting at the focus, respectively. γ reflects the surface-state lifetime, α reflects the scattering amplitude, and Δ_s is the hybridization energy of the adatom and surface state. The error bars are standard deviation (hereinafter the same).

e	0.4	0.45	0.5	0.6	0.65	0.76
δ_c (π)	1.07±0.01	1.06±0.01	1.10±0.01	1.08±0.02	1.25±0.02	1.17±0.02
δ_f (π)	1.42 ±0.01	1.34±0.01	1.35±0.01	1.37±0.01	0.84±0.01	1.18±0.01
γ (meV)	8.8±0.2	8.9±0.2	8.4±0.3	8.2±0.5	11.7±0.8	12.0±1.0
$\alpha\rho_s$	0.33±0.01	0.29±0.01	0.15±0.01	0.37±0.01	0.27±0.01	0.25±0.01
$\Delta_s\rho_s$	0.23±0.01	0.20±0.01	0.15±0.01	0.13±0.01	0.22±0.01	0.22±0.01

Supplementary Note 9: Fitting parameters and quantization conditions for changing the value of *a*

Similarily, we used Eq. (2) to fit the experimental results of Fig. 4 in main text. The values obtained for these parameters appear in Supplementary Table 2. The fitted values are consistent with those listed in Supplementary Table 1.

Supplementary Table 2 Fitting results obtained for increasing values of *a* (*e* = 0.5).

a (nm)	5.7	6.8	7.6	8.6	9.4	10.4
δ_c (π)	1.05±0.01	1.05±0.01	1.10±0.01	1.15±0.01	1.22±0.01	1.17±0.01
δ_f (π)	1.56 ±0.01	1.64±0.01	1.35±0.01	1.31±0.01	1.19±0.01	1.22±0.01
γ (meV)	7.3±0.4	7.0±0.3	8.4±0.2	7.8±0.2	9.1±0.3	8.2±0.3
$\alpha\rho_s$	0.28±0.01	0.27±0.01	0.29±0.01	0.24±0.01	0.28±0.01	0.25±0.01
$\Delta_s\rho_s$	0.13±0.01	0.13±0.01	0.19±0.01	0.17±0.01	0.19±0.01	0.17±0.01

Although the peaks shift left with increasing values of *a* (Fig. 4), the values of *ka* remain almost the same for the different peaks (labeled by 1, 2, 3, …), respectively. Supplementary Fig. 11 shows the values of *ka* for different peaks versus *a*. Stars are the experimental results of the peaks in Fig. 4, where the red stars stand for the central peaks and the black stars represent the other peaks. All peaks agree well with the theoretically derived first (blue dash lines) and second (yellow dotted lines) quantization conditions. Remarkably, the central peaks appear where these two theoretical derived conditions coincide with each other, indicating that both quantization conditions are of critical importance. Note that the peaks labelled 7 and 8 for *a* = 8.6 nm have almost the same intensity, which leads to a discrepancy in the central peak counting. In addition, peaks 1 and 2 are not observed due to the fast decay at small values of *k*. The one-to-one correspondence between the peak positions and the two quantized conditions once again

supports the validity of the quantum interference model.

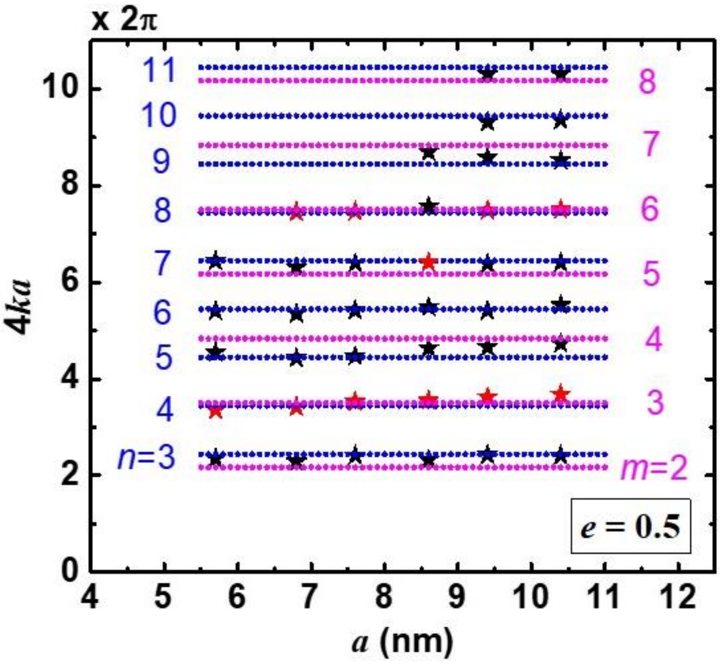

Supplementary Figure 11 | Quantization conditions *versus a* value. Comparison of the experimentally determined peak positions (red stars are the central peaks and the dark blue stars are the other peaks) and the theoretically obtained quantized conditions. Blue lines marked by *n* are the condition mentioned in Eq. (3), and magenta dotted lines marked by *m* are the conditions described with Eq. (4).

Supplementary Note 10: Decay behavior check

To quantify the decay behavior of the transfer function, we used $\frac{\Delta(dI/dV)_{\mathrm{rf}}}{(dI/dV)_{\mathrm{lf}}} = C_2 A_2 \cos\left[2(1+e)ka+\delta_2\right] + C_3 A_3 \cos(4ka+\delta_3)$ to fit the experimental results of Fig. 4. C_2 and C_3 are fitting parameters that describe the oscillating amplitudes. Supplementary Fig. 12 shows results for amplitudes C_2 and C_3 versus a. We find that the logarithmic values of C_2 and C_3 decay linearly with increasing a. C_3 decays faster

than C_2, and the slope ratio of C_3/C_2 is 1.2 ± 0.1, which is consistent with $2/(1+e)$ for $e=0.5$.

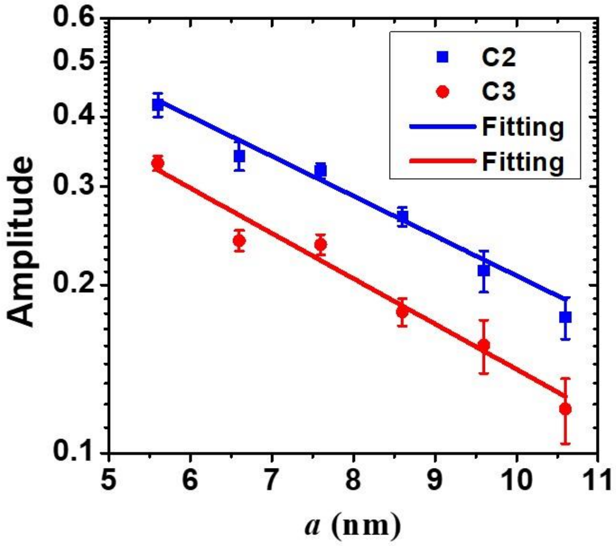

Supplementary Figure 12 | Logarithmic values of amplitudes C_2 and C_3 versus *a*. The decays show a linear behavior on the logarithmic scale.

Supplementary Note 11: Comparison of the computed the *dI/dV* spectra at the right focus with the one obtained experimentally

Our study reveals that the transmission process of the quantum mirage is dominated by path 2 (magenta triangle in Fig. 3a) and path 3 (blue quadrangle in Fig. 3a). With the computed transfer function, we can essentially reproduce the mirage (the spectrum at the right focus), see Supplementary Fig. 13 for three different corrals. The parametrs used in the transfer function are taken from the corresponding mean values of Supplementary Table 1. Note the calculations were made for an ideally positioned corral. The slight deviation may originate from non-perfect positioning of the adatoms used for the corral construction.

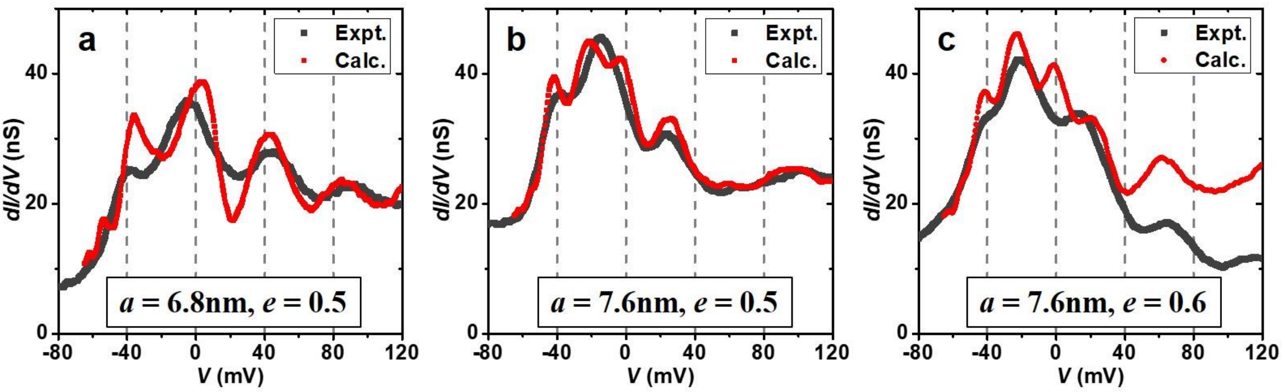

Supplementary Figure 13 | Comparison of the calculated spectra (red symbols) with the experimentally obtained one (black symbols) at right focus. The agreement indicates that the experimental spectra can be calculated with the theoretically derived transfer function, the spectra of the Fe adatom placed at left focus and the empty right focus.

Supplementary Note 12: Pseudo OR logic gate

When the input and output are swopped, the confocal EQC can also be used to construct another basic pseudo logic operation—the OR gate as illustrated in Supplementary Fig. 14. In it, we used both foci B and C as the inputs and the joint focus A as the output. When both B and C are empty, namely "0" (Supplementary Fig. 14a), the corresponding *dI/dV* at joint focus A (Supplementary Fig. 14b) has a low intensity of 29.2 nS. When one of the foci B and C are configured as "1" (Supplementary Figs. 14c & 14e), the output at A has an intensity of ~34 nS (Supplementary Figs. 14d & 14f). When both foci B and C are configured as "1" (Supplementary Fig. 14g), the output at A has the intensity of 38.2 nS. Even though this intensity is slightly higher than that obtained when either B or C is configured as "1", the device functions as an OR logic

gate when the threshold is set to ~32 nS. (Note that if the threshold is set to ~36 nS, this logic operation can be viewed as an AND gate). In the above we used the energy at the constructive phase of interference. When the energy is chosen to be at the destructive phase, a pseudo NOR logic gate (not shown) can also form, similar to a pseudo NOT logic gate.

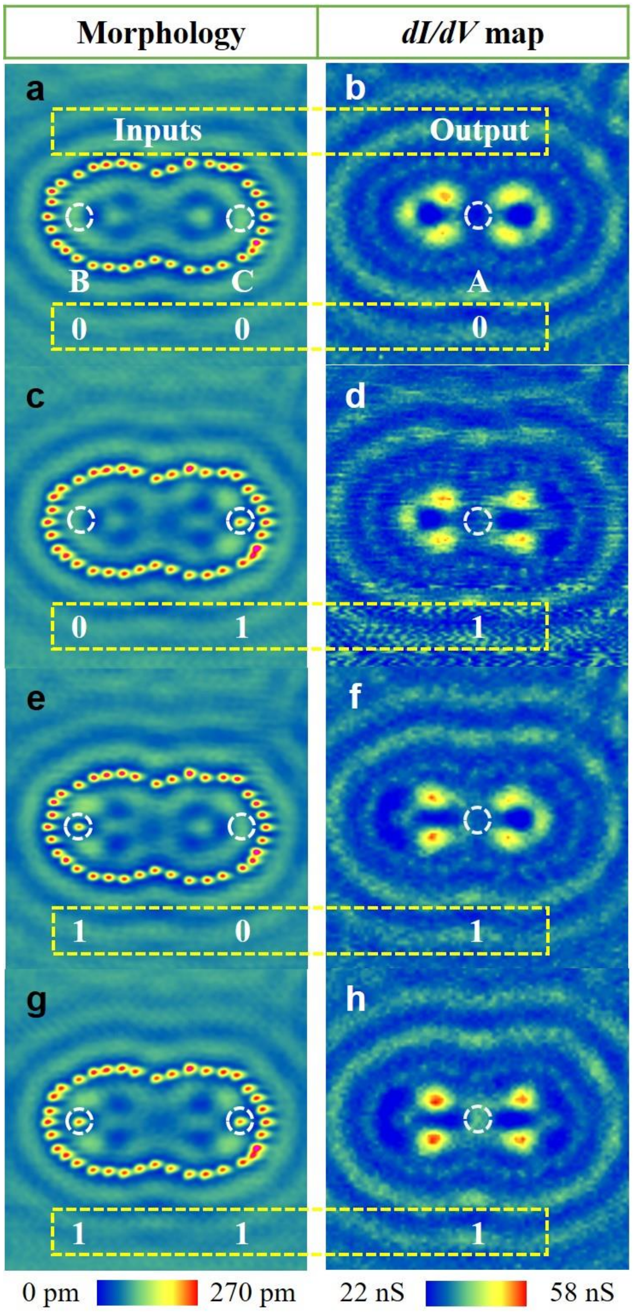

Supplementary Figure 14 | Pseudo OR logic gate. The left column shows the

topographical images (**a**, **c**, **e**, **g**) and the right column is the corresponding *dI/dV* maps with bias voltage +12 mV (**b**, **d**, **f**, **h**). Both foci B and C are inputs and the joint focus A is used as the output. If one or more of the left and right foci are occupied, the output is 1. Otherwise, the output is 0. The EQCs used to build the confocal EQC have a = 6.6 nm and e = 0.6.

Supplementary Table 3 Values of inputs and outputs for pseudo OR logic gate. The bias voltage for *dI/dV* map is −12 mV.

**Inputs**		**Output**
**A (Atom occupation)**	**B (Atom occupation)**	**C (*dI/dV* in nS)**
0	0	29.2±1.3
0	1	33.7±1.2
1	0	34.6±1.9
1	1	38.2±1.7

Supplementary References

1. Madhavan, V., Chen, W., Jamneala, T., Crommie, M. F. & Wingreen, N. S. Local spectroscopy of a Kondo impurity: Co on Au(111). *Phys. Rev. B* **64**, 165412 (2001).
2. Olsson, F. E., Persson, M., Borisov, A. G., Gauyacq, J. P., Lagoute, J. & Fölsch, S. Localization of the Cu(111) surface state by single Cu adatoms. *Phys. Rev. Lett.* **93**, 206803 (2004).
3. Limot, L., Pehlke, E., Kröger, J. & Berndt, R. Surface-state localization at adatoms. *Phys. Rev. Lett.* **94**, 036805 (2005).
4. Lounis, S., Mavropoulos, P., Dederichs, P. H. & Blügel, S. Surface-state scattering by adatoms on noble metals: Ab initio calculations using the Korringa-Kohn-Rostoker Green function method. *Phys. Rev. B* **73**, 195421 (2006).
5. Lazarovits, B., Szunyogh, L. & Weinberger, P. Spin-polarized surface states close to adatoms on Cu(111). *Phys. Rev. B* **73**, 045430 (2006).
6. Limot, L., Kröger, J., Berndt, R., Garcia-Lekue, A. & Hofer, W. A. Atom Transfer and Single-Adatom Contacts. *Phys. Rev. Lett.* **94**, 126102 (2005).
7. Manoharan, H. C., Lutz, C. P. & Eigler, D. M. Quantum mirages formed by coherent projection of electronic structure. *Nature* **403**, 512-515 (2000).
