# Supplementary material for: Kondo-free mirages in elliptical quantum corrals
Source: Nat Commun. 2020 Mar 16;11:1400. doi: 10.1038/s41467-020-15137-8 (PMC7075878; doi:10.1038/s41467-020-15137-8)
Supplement: Supplementary file 2 — Peer Review File [file 41467_2020_15137_MOESM2_ESM.docx]

REVIEWERS' COMMENTS:

Reviewer #1 (Remarks to the Author):

The manuscript from Li et al. reports a scanning tunneling microscopy and spectroscopy investigation on elliptical quantum corrals realized on Ag(111) by means of atomic manipulation of Fe adatoms. By placing an Fe atom at one of the focus of the elliptical corral, the authors investigated the perturbation of the surface local density of state at the other focus. Using a Green's functions based analytical approach from the literature [see Refs. 5 and 12 for example], they model with great precision the scattering of electrons within the corral and account for the experimental transfer function between the foci. Finally, they proposed a conceptual use of the inversion effect on the differential conductance to realize atomic scale logic gates whose input (or output) can be controlled (or detected) by atomic manipulation (or differential conductance readout).

The quality of both the experiment and the analysis is very high and the last section contains an original proposition that would surely stimulate further thoughts and researches in the field. Beside the positive notes, I have three major concerns related to the publication of this paper in Nature Communication.

1) As the title suggests, the author claims a Kondo-free quantum mirage occurring in their system. In my opinion, this claim is misleading for the following reasons:

- in order to produce a mirage, the property of an object placed in one focus should be observed at the other one.

- in the original work of Manoharan et al. [Ref. 2], a mirage of the Kondo zero-bias anomaly of the adatom at the focus could be detected at the other.

- in the present work, the relevant spectroscopic feature at the adatom position is the inversion peak-valley-peak to valley-peak-valley effect. However, the transmission of this effect looks more complicated than a simple refocusing effect from one focus to the other. For example, as shown in Fig. 1j, the dI/dV spectra look very different from one focus to the other. In addition, in the elliptical quantum corral two other dI/dV maxima appear along the minor axis. It is therefore difficult to understand what is the effective mirage occurring in this system, and what is instead the overall quantum interference occurring within the corral, which can also originate series of maxima and minima in the dI/dV. I strongly recommend to show further analysis of dI/dV spectra in high symmetry point of the ellipse to disentangle the quantum interference effect from the expected quantum mirage.

2) Although the quality of the data is indeed high, the analysis follows a well known method [Ref. 5 and 12 and others in the text] and essentially confirms the expected behavior for electron scattering in a 2D quantum well potential. Therefore, the original point of the paper is mostly the attempt of showing the observation of a quantum mirage (which I have questioned already in point 1) and the proposition of using this system as a conceptual logic gate

3) Related to this last point, I do not deny the originality of the author proposition, especially for the two-lobe corral shown in Fig. S12. However, the authors should provide more quantitative assessment of the quality of the created gate. As discussed for example in Nanoscale, 2015, 7, 8261-8283, typical values of the on-off signal in real devices is of the order of 10^4 - 10^7. Here the authors show logic thresholds that barely reach a ratio of 2. I recommend the authors to provide more detailed comments on the related value of their thresholds, and also to discuss the possibility of improving their on-off ratio to reach values that approaches those of real applications.

Other minor points:

- Page 3, lines 60-70: the explanation of the inversion effect is not very clear. A reader needs to check more details in Ref. 22 to better understand the origin of this effect. As the inversion effect is a main point related to the quantum mirage, I recommend to improving this section.

- Page 5, lines 101-109: I would recommend the authors to explicitly mention that the voltage axis of the dI/dV is that converted in k wavenumber in Fig. 2 using the dispersion relation. I initially got confused as k-vector plots related to quasi-particle interference are often associated to fourier transform of the dI/dV map, whereas in the present case this is a direct transformation of the point spectra at the foci.

- Page 8, lines 159-162: I suggest to properly define the parameter gamma, which is related to the imaginary part

of the self energy. This will help readers that are not familiar with Green's function theory to follow the results of their approach

Typos:

- Page 3, line 50: density states -> density of states

Reviewer #2 (Remarks to the Author):

In this manuscript the authors discuss mirage effects in quantum corrals that persist in energy ranges beyond the immediate vicinity of the Fermi energy. An experimental study and a corresponding theoretical analysis are presented. Overall, I think the results are important.

However, the paper has a large number of English usage issues, particularly in the introductory parts. I disagree with the opening statement of the second paragraph of the paper: “Different theories reproduce the mirage effect, but the mechanism is largely elusive.” The existing (published) theory does not suggest there is anything elusive about the mirage—it is the result of surface state electrons scattering within an elliptical corral, both off the boundaries and any impurity at a focus. From the point-of-view of the scattered electrons, there is no restriction on the energy range over which the mirage can be observed, and its character will in general depend on the energy range over which the surface states are perturbed by the impurity at the focus.

In this work, the authors report an interesting eccentricity dependence of the mirage effect, which they describe in the dI/dV spatial images and on-focus spectroscopy. To the best of my knowledge, the eccentricity-dependent mirage effects that have been uncovered are new. The model introduced in Eq.(1) takes into account multiple scattering events within the corral, and includes the k-dependent phase coherence length, which is an important part of the theoretical analysis of this work, as seen in Eq.(2).

The theoretical analysis does not appear to incorporate the bulk states, aside possibly from the k-dependence of the phase coherence length, which is shorter when impurities are present on the surface (a physical interpretation that may be consistent with loss of electron amplitude to bulk states when surface impurities are present). From the analysis, it appears this might be a complementary way to the imaginary part of the phase shift used by some of the scattering theories employed for prior studies of the quantum corrals.

The final part of the paper, which discusses logic gates, seems like it may be unrealistic for quantum corrals made from adatoms on noble metal surfaces. However, many of these ideas are transferrable to optical devices in which light is trapped in a two-dimensional dielectric medium. Since the effects are fundamentally related to wave phenomena (and not quantum statistics, for example) there may be light-based applications of these ideas down the road.

I recommend publication after the authors have addressed the points raised above.

Reviewer #3 (Remarks to the Author):

The manuscript “Kondo-free quantum mirage: mechanism and manipulation” by Qili Li et al. describes an in depth investigation of information transfer using artificially constructed quantum corrals with properties which are tailored to achieve the propagation of spectroscopic information from an atom to a remote position via the electronic surface state of a metal substrate.

The main findings are:

(i) that the information transfer is feasible without the need of a specific spectroscopic signature, as, e.g., that of a Kondo atom which has been experimentally done in the past.

And (ii) this information transfer enables the realization of proofs of principle of novel concepts for information processing in the form of logic gates.

The topic and content of the manuscript is of very high interest to the general science community warranting the requirements for a publication in Nature Communications. The experimental data are of high quality, and their interpretations using the simulations are very convincing.

I only have to criticize the presentation of the results, i.e. in particular the English language, which needs to be improved. Moreover, I would propose to already specify the substrate used for the study in Line 46 on page 3.

**Response to Reviewer’s comments**

We thank the three reviewers for their constructive comments/suggestions. We provide our point-by-point responses to all comments/concerns below in blue font. The changes to the main text (other than trivial typos) are highlighted in red.

*Reviewer #1 (Remarks to the Author):*

*The manuscript from Li et al. reports a scanning tunneling microscopy and spectroscopy investigation on elliptical quantum corrals realized on Ag(111) by means of atomic manipulation of Fe adatoms. By placing an Fe atom at one of the focus of the elliptical corral, the authors investigated the perturbation of the surface local density of state at the other focus. Using a Green's functions based analytical approach from the literature [see Refs. 5 and 12 for example], they model with great precision the scattering of electrons within the corral and account for the experimental transfer function between the foci. Finally, they proposed a conceptual use of the inversion effect on the differential conductance to realize atomic scale logic gates whose input (or output) can be controlled (or detected) by atomic manipulation (or differential conductance readout).*

*The quality of both the experiment and the analysis is very high and the last section contains an original proposition that would surely stimulate further thoughts and researches in the field. Beside the positive notes, I have three major concerns related to the publication of this paper in Nature Communication.*

*1) As the title suggests, the author claims a Kondo-free quantum mirage occurring in their system. In my opinion, this claim is misleading for the following reasons:*

*- in order to produce a mirage, the property of an object placed in one focus should be observed at the other one.*

*- in the original work of Manoharan et al. [Ref. 2], a mirage of the Kondo zero-bias anomaly of the adatom at the focus could be detected at the other.*

*- in the present work, the relevant spectroscopic feature at the adatom position is the inversion peak-valley-peak to valley-peak-valley effect. However, the transmission of this effect looks more complicated than a simple refocusing effect from one focus to the other. For example, as shown in Fig. 1j, the dI/dV spectra look very different from one focus to the other. In addition, in the elliptical quantum corral two other dI/dV maxima appear along the minor axis. It is therefore difficult to understand what is the effective mirage occurring in this system, and what is instead the overall quantum interference occurring within the corral, which can also originate series of maxima and minima in the dI/dV. I strongly recommend to show further analysis of dI/dV spectra in high symmetry point of the ellipse to disentangle the quantum interference effect from the expected quantum mirage.*

Response to #1-1

We sincerely thank Reviewer #1 for summarizing our main findings and for the positive comments on the quality and the novelty of our work.

Reviewer #1 raised an interesting point, the comparison our work with the pioneering work by Manoharan *et al.* [Ref. 2]. Manoharan *et al*., reported that the Kondo resonance (zero-bias anomaly) of a Co adatom placed at one focal point of an elliptical corral on a Cu(111) surface can be imaged at the other focal point. The mirage is demonstrated in a narrow energy window near the Fermi energy. Our work shows that the mirage of Fe or Ag adatoms can be realized over a wide energy range on a Ag(111) surface. As the wavelength of the surface electron is energy dependent, the transfer function is also energy dependent (as shown in Figs. 2 and 4). It is indeed different than the transfer function reported by Manoharan *et al,* which is almost a constant since it is only for a narrow energy range near the Fermi energy. However, our study reveals that the transmission process is not complicated, in that with the computed transfer function, we can essentially reproduce the mirage (the spectrum at the right focus), see Fig. R1 for three different corrals. We provide a short discussion about this in the revised version (L. 193 and L. 197 in main text, and Note 11 of Supplementary Information).

Fig. R1 Comparison of the calculated spectra (red symbols) with the experimentally obtained one (black symbols) at right focus. The agreement indicates that the experimental spectra can be calculated from the theoretically-derived transfer function provided that the spectra of the Fe adatom placed at the left focus and the empty right focus are known. Note that the calculations were made for ideally positioned corrals. The slight deviation may originate from the non-perfect positioning of the adatoms used in the corral construction.

Review #1 also asked why the *dI/dV* map in Fig. 1j looks different from focus to the other. This can be understood from a diffraction point of view. In the work by Manoharan *et al*., the quantum mirage is realized near the Fermi energy on a Cu(111) surface. The wavelength of the Cu(111) surface state at the Fermi level is ~3 nm. The resolution limit (~0.6 wavelength) is ~1.8 nm, which is close to the size of the Co adatom. Thus, the distortion is not visualized and the image looks similar to the object. In our case, the data shown in Fig. 1j is taken at −6 meV on an Ag(111) surface (Note that the data of the mirage in Fig. 1j was chosen due to its high intensity). The corresponding wavelength is ~7.8 nm while the Fe atom appears ~1 nm in diameter at the left focus. Thus, we are imaging a subwavelength object; the image appears larger than the object and also somewhat distorted due to the resolution limit. When we increase the energy for imaging, the distortion reduces, as shown in Figs. S3b and S3h. We provide a note about this in the revised version (L. 90 in main text).

In our supplementary information, we already showed that when the adatom is placed out of the focus position, the mirage effect disappeared (Fig. S3). Upon the reviewer’s suggestion, we also performed the *dI/dV* map analysis in high symmetry points of the ellipse. More specifically, we placed the adatom at different points either at the long axis or the short axis and made spectroscopy measurements. The results are shown below in Fig. R2. From the figure, we find that quantum mirage can only be obtained when the adatom is placed at the focus position (Figs. R2a and R2b). When the adatom is not placed at the focus point, even though it is placed at high symmetry points of the ellipse, it does not show any apparent feature either at the focus point or at its symmetrical point (Figs. R2c-R2h). This once again confirms the importance of the focal point and that the effect that we observed is indeed a quantum mirage. We also added this discussion in Note 3 of Supplementary Information.

Fig. R2 Comparisons of *dI/dV* map for adatoms placed at different locations of an EQC with *e* = 0.5, *a* = 7.8 nm. The origin of coordinates is the ellipse center. The red ellipses, yellow crosses and purple circles mark the positions of the quantum corrals, the foci and the adatoms and their corresponding symmetrical points, respectively. The *dI/dV* maps were measured at 10 mV.

*2) Although the quality of the data is indeed high, the analysis follows a well known method [Ref. 5 and 12 and others in the text] and essentially confirms the expected behavior for electron scattering in a 2D quantum well potential. Therefore, the original point of the paper is mostly the attempt of showing the observation of a quantum mirage (which I have questioned already in point 1) and the proposition of using this system as a conceptual logic gate.*

Response to #1-2

We thank the reviewer for appreciating our data quality. But we only partially agree on the comment about our analysis. We indeed followed a similar approach as used in Refs. 5 and 12 as explicitly mentioned in the text. However, our result is different, in particular, in the eccentricity-dependence. In the abstract of Ref. 5, the authors pointed out that a quantum mirage is *independen*t of the size and shape of the ellipse. Ref. 12 essentially followed the approach of Ref. 5 but extended the discussion to the vibrational modes. Our experiments show that the quantum mirages clearly are size and shape dependent. These dependences cannot be explained by Refs. 5 or 12 but requires our new analysis.

*3) Related to this last point, I do not deny the originality of the author proposition, especially for the two-lobe corral shown in Fig. S12. However, the authors should provide more quantitative assessment of the quality of the created gate. As discussed for example in Nanoscale, 2015, 7, 8261-8283, typical values of the on-off signal in real devices is of the order of 10^4 - 10^7. Here the authors show logic thresholds that barely reach a ratio of 2. I recommend the authors to provide more detailed comments on the related value of their thresholds, and also to discuss the possibility of improving their on-off ratio to reach values that approaches those of real applications.*

Response to #1-3

We thank the reviewer for this suggestion. We added a discussion in L. 232 in main text, as follows:

“Here, as a prototype device, the logic threshold of the on-off signal of ~1.5 is demonstrated. It is somewhat less than that of the real devices [Nanoscale, 2015, 7, 8261-8283]. This ratio, however, can be improved via several approaches. For instance, in our work, the bulk state contributes as a constant background that significantly reduces the on-off ratio. Thus, a higher ratio can be achieved if the experiment is performed on a system with a reduced or even vanishing bulk state. Besides, as above discussed, the amplitude of the transfer function depends on the DOS of the surface state and the hybridization energy. When the intensities of these two terms are increased, a higher ratio is also expected.”

*Other minor points:*

*- Page 3, lines 60-70: the explanation of the inversion effect is not very clear. A reader needs to check more details in Ref. 22 to better understand the origin of this effect. As the inversion effect is a main point related to the quantum mirage, I recommend to improving this section.*

Response to #1-4

We followed the suggestion and revised the manuscript accordingly (L. 66 in main text).

*- Page 5, lines 101-109: I would recommend the authors to explicitly mention that the voltage axis of the dI/dV is that converted in k wavenumber in Fig. 2 using the dispersion relation. I initially got confused as k-vector plots related to quasi-particle interference are often associated to fourier transform of the dI/dV map, whereas in the present case this is a direct transformation of the point spectra at the foci.*

Response to #1-5

We followed the suggestion and mentioned in the caption of Fig. 2 how the values of the wavenumber (*k*) are obtained.

*- Page 8, lines 159-162: I suggest to properly define the parameter gamma, which is related to the imaginary part of the self energy. This will help readers that are not familiar with Green's function theory to follow the results of their approach*

Response to #1-6

We followed the suggestion and explained the physical meaning of gamma in the revised manuscript (L. 165 in main text).

*Typos:*

*- Page 3, line 50: density states -> density of states*

Response to #1-7

We thank Reviewer #1 for pointing out this typo. We corrected it and further crosschecked the manuscript throughout.

*Reviewer #2 (Remarks to the Author):*

*In this manuscript the authors discuss mirage effects in quantum corrals that persist in energy ranges beyond the immediate vicinity of the Fermi energy. An experimental study and a corresponding theoretical analysis are presented. Overall, I think the results are important.*

*However, the paper has a large number of English usage issues, particularly in the introductory parts. I disagree with the opening statement of the second paragraph of the paper: “Different theories reproduce the mirage effect, but the mechanism is largely elusive.” The existing (published) theory does not suggest there is anything elusive about the mirage—it is the result of surface state electrons scattering within an elliptical corral, both off the boundaries and any impurity at a focus. From the point-of-view of the scattered electrons, there is no restriction on the energy range over which the mirage can be observed, and its character will in general depend on the energy range over which the surface states are perturbed by the impurity at the focus.*

Response to #2-1

We thank Reviewer #2 for the positive review and recognizing the importance of our results.

We agree with Reviewer #2 that the quantum mirage can be understood as the result of surface state electrons scattering within an elliptical corral, both off the boundaries and any impurity at a focus. However, this is a general understanding. It does not tell us under what conditions the quantum mirage can form. In fact, as discussed in our introduction, different groups have very different opinions on the formation conditions. Beside, as the reviewer points out in the next paragraph, whether and how the quantum mirage depends on the eccentricity is also an unresolved issue. Thus, we changed the opening sentence to “Different theories [5-14] reproduce the mirage effect but point out different key factors for the formation mechanism.”

*In this work, the authors report an interesting eccentricity dependence of the mirage effect, which they describe in the dI/dV spatial images and on-focus spectroscopy. To the best of my knowledge, the eccentricity-dependent mirage effects that have been uncovered are new. The model introduced in Eq.(1) takes into account multiple scattering events within the corral, and includes the k-dependent phase coherence length, which is an important part of the theoretical analysis of this work, as seen in Eq.(2).*

*The theoretical analysis does not appear to incorporate the bulk states, aside possibly from the k-dependence of the phase coherence length, which is shorter when impurities are present on the surface (a physical interpretation that may be consistent with loss of electron amplitude to bulk states when surface impurities are present). From the analysis, it appears this might be a complementary way to the imaginary part of the phase shift used by some of the scattering theories employed for prior studies of the quantum corrals.*

Response to #2-2

We thank Review #2 for this suggestion. We added a note to the alternative approach that includes bulk states (L. 396 in main text).

*The final part of the paper, which discusses logic gates, seems like it may be unrealistic for quantum corrals made from adatoms on noble metal surfaces. However, many of these ideas are transferrable to optical devices in which light is trapped in a two-dimensional dielectric medium. Since the effects are fundamentally related to wave phenomena (and not quantum statistics, for example) there may be light-based applications of these ideas down the road.*

Response to #2-3

We thank Reviewer #2 for this suggestion. We added a short discussion about this in the revised manuscript in L. 249 in main text, as follows:

“In addition, in our logic gates, essentially only the wave property of the electrons is used. This kind of wave property, such as the standing wave inside a corral, is also realized in optical systems [ACS Nano 2009, 3, 615-620]. Thus, the demonstrated concepts are expected to be transferrable to other wave-based devices, such as optical or magnon devices [Laser & Photonics Reviews 7, 882-900 (2013), Nat. Phys. 11, 453 (2015)].”

*I recommend publication after the authors have addressed the points raised above.*

Response to #2-4

We sincerely thank the reviewer for the recommendation. We hope that the reviewer will find our responses are satisfying.

*Reviewer #3 (Remarks to the Author):*

*The manuscript “Kondo-free quantum mirage: mechanism and manipulation” by Qili Li et al. describes an in depth investigation of information transfer using artificially constructed quantum corrals with properties which are tailored to achieve the propagation of spectroscopic information from an atom to a remote position via the electronic surface state of a metal substrate.*

*The main findings are:*

*(i) that the information transfer is feasible without the need of a specific spectroscopic signature, as, e.g., that of a Kondo atom which has been experimentally done in the past.*

*And (ii) this information transfer enables the realization of proofs of principle of novel concepts for information processing in the form of logic gates.*

*The topic and content of the manuscript is of very high interest to the general science community warranting the requirements for a publication in Nature Communications. The experimental data are of high quality, and their interpretations using the simulations are very convincing.*

*I only have to criticize the presentation of the results, i.e. in particular the English language, which needs to be improved. Moreover, I would propose to already specify the substrate used for the study in Line 46 on page 3.*

Response to #3

We sincerely thank Reviewer #3 for summarizing our main findings and the recommendation. We followed the suggestion and had a native English speaker help improve the presentation.

REVIEWERS' COMMENTS:

Reviewer #1 (Remarks to the Author):

The authors have reviewed the manuscript following the suggestions of all referees. Therefore, in my opinion the paper is now suitable for publication.

I just have a final minor remark. In the revised supplementary material, at page 20, the authors state that "the transmission process of the quantum mirage is not complicated". Do they mean "not complex" in its nature, or "not complicated" in its description using the proposed formalism?

Reviewer #2 (Remarks to the Author):

I am satisfied with the author's response to the reviewer comments and now recommend the paper for publication.

**Response to Reviewer’s comments**

*Reviewer #1 (Remarks to the Author):*

*The authors have reviewed the manuscript following the suggestions of all referees. Therefore, in my opinion the paper is now suitable for publication.*

*I just have a final minor remark. In the revised supplementary material, at page 20, the authors state that "the transmission process of the quantum mirage is not complicated". Do they mean "not complex" in its nature, or "not complicated" in its description using the proposed formalism?*

Response to #1

We sincerely thank Reviewer #1 for the recommendation and the interesting remark. We feel that the wording of “complicated” is subjective. Different people may have different opinions. Thus, we replaced it with an objective description. That is, “Our study reveals that the transmission process of the quantum mirage is dominated by path 2 (magenta triangle in Fig. 3a) and path 3 (blue quadrangle in Fig. 3a)”.

*Reviewer #2 (Remarks to the Author):*

*I am satisfied with the author's response to the reviewer comments and now recommend the paper for publication.*

Response to #2

We sincerely thank Reviewer #2 for the recommendation.
